# The knob protein KAHRP assembles into a ring-shaped structure that underpins virulence complex assembly

**DOI:** 10.1371/journal.ppat.1007761

**Published:** 2019-05-09

**Authors:** Oliver Looker, Adam J. Blanch, Boyin Liu, Juan Nunez-Iglesias, Paul J. McMillan, Leann Tilley, Matthew W. A. Dixon

**Affiliations:** 1 Department of Biochemistry and Molecular Biology, Bio21 Molecular Science and Biotechnology Institute, University of Melbourne, Parkville, Australia; 2 Biological Optical Microscopy Platform, Bio21 Molecular Science and Biotechnology Institute, University of Melbourne, Parkville, Australia; Francis Crick Institute, UNITED KINGDOM

## Abstract

*Plasmodium falciparum* mediates adhesion of infected red blood cells (RBCs) to blood vessel walls by assembling a multi-protein complex at the RBC surface. This virulence-mediating structure, called the knob, acts as a scaffold for the presentation of the major virulence antigen, *P*. *falciparum* Erythrocyte Membrane Protein-1 (PfEMP1). In this work we developed correlative STochastic Optical Reconstruction Microscopy–Scanning Electron Microscopy (STORM-SEM) to spatially and temporally map the delivery of the knob-associated histidine-rich protein (KAHRP) and PfEMP1 to the RBC membrane skeleton. We show that KAHRP is delivered as individual modules that assemble *in situ*, giving a ring-shaped fluorescence profile around a dimpled disk that can be visualized by SEM. Electron tomography of negatively-stained membranes reveals a previously observed spiral scaffold underpinning the assembled knobs. Truncation of the C-terminal region of KAHRP leads to loss of the ring structures, disruption of the raised disks and aberrant formation of the spiral scaffold, pointing to a critical role for KAHRP in assembling the physical knob structure. We show that host cell actin remodeling plays an important role in assembly of the virulence complex, with cytochalasin D blocking knob assembly. Additionally, PfEMP1 appears to be delivered to the RBC membrane, then inserted laterally into knob structures.

## Author summary

The human malaria parasite *Plasmodium falciparum* causes severe disease, which is initiated by the adhesion of parasite-infected RBCs to receptors on the walls of the host’s capillaries. Adhesion is mediated by a structure called the knob, which acts as a scaffold for the presentation of the virulence protein, *P*. *falciparum* erythrocyte membrane protein-1 (PfEMP1). In this work we investigate the assembly of this complex at different stages of parasite development using a multimodal imaging approach that combines *d*STORM localization microscopy and scanning electron microscopy (STORM-SEM). We show that the knob-associated histidine-rich protein (KAHRP) is delivered to the RBC membrane skeleton as individual protein modules that assemble into a ring-shaped complex. We provide evidence that host cell remodeling, driven by association of KAHRP with spectrin and the reorganization of actin, is required for assembly of the ring complex, which in turn supports a spiral scaffold that is required for correct knob morphology. Finally, we provide evidence that PfEMP1 is delivered to the RBC membrane before associating with knob complexes.

## Introduction

Malaria is a devastating human disease that affects more than a quarter of the world’s population and kills more than 400,000 people annually [1]. The majority of these deaths are caused by infection with *Plasmodium falciparum*, with children from sub-Saharan Africa most at risk. *P*. *falciparum*-infected red blood cells (RBCs) sequester within the microvasculature of the host, via a process called cytoadhesion. Adhesion removes the infected RBCs from the circulation, thereby avoiding clearance in the spleen, and thus contributing to parasite virulence. Moreover, adhesion of infected RBCs to brain venule endothelial cells or to placental syncytiotrophoblasts can lead to severe complications, known as cerebral and placental malaria, respectively [2, 3]. Adhesion of infected RBCs is mediated by a family of proteins called *P*. *falciparum* erythrocyte membrane protein-1 (PfEMP1). PfEMP1 is encoded by the ~60-member *var* gene family [4, 5]. Once inserted into the RBC membrane, PfEMP1 can bind to endothelial cell receptors such as Endothelial Protein C Receptor (EPCR), Cluster Determinant 36 (CD36), Intercellular Adhesion Molecule-1 (ICAM), and glycosaminoglycans, driving parasite sequestration [6, 7].

Central to the parasite’s ability to cytoadhere is the formation of nano-scale protrusions (called knobs) at the RBC membrane that act as platforms for the presentation of PfEMP1. The knob-associated histidine-rich protein (KAHRP) is a component of these structures and is essential for their formation [8]. In the absence of knobs, infected RBCs are unable to adhere to endothelial receptors under physiologically-relevant flow conditions [8]. A recent study suggested that the knob comprises a cone-shaped coat of electron-dense KAHRP molecules, underpinned by a spiral structure (of unknown composition) and connected by multiple links to the actin-spectrin meshwork of the RBC membrane skeleton [9]. Work with recombinant proteins and with parasite mutants expressing truncated KAHRP has shown that the C-terminal region of KAHRP contains a sequence that drives high affinity binding to spectrin [10, 11] and is critical for the formation of knobs with normal morphology [12].

Despite the important contribution of the virulence complex to severe disease [13], it remains unclear how knobs are assembled at the RBC membrane and how PfEMP1 is loaded into the knobs and presented at the surface. To understand the processes underpinning the assembly of the virulence complex, it is important to determine the locations of individual components with the highest possible spatial resolution, and to place this spatial information in the context of the physical cellular structures. Here we combine direct STochastic Optical Reconstruction Microscopy (*d*STORM) localization microscopy with scanning electron microscopy (SEM) to image the assembly of modular units of KAHRP and the delivery of PfEMP1 to the knobs. We show that the spectrin-actin meshwork plays an important role in marshalling knob formation and that sequences within the C-terminal region of KAHRP control assembly into rings and are essential for maintaining the structure of the spiral knob scaffold. Loading of PfEMP1 into the fully formed knob structures appears to occur by lateral transfer.

## Results

### KAHRP forms ring-shaped complexes that associate with the RBC membrane skeleton

**Fig 1:**
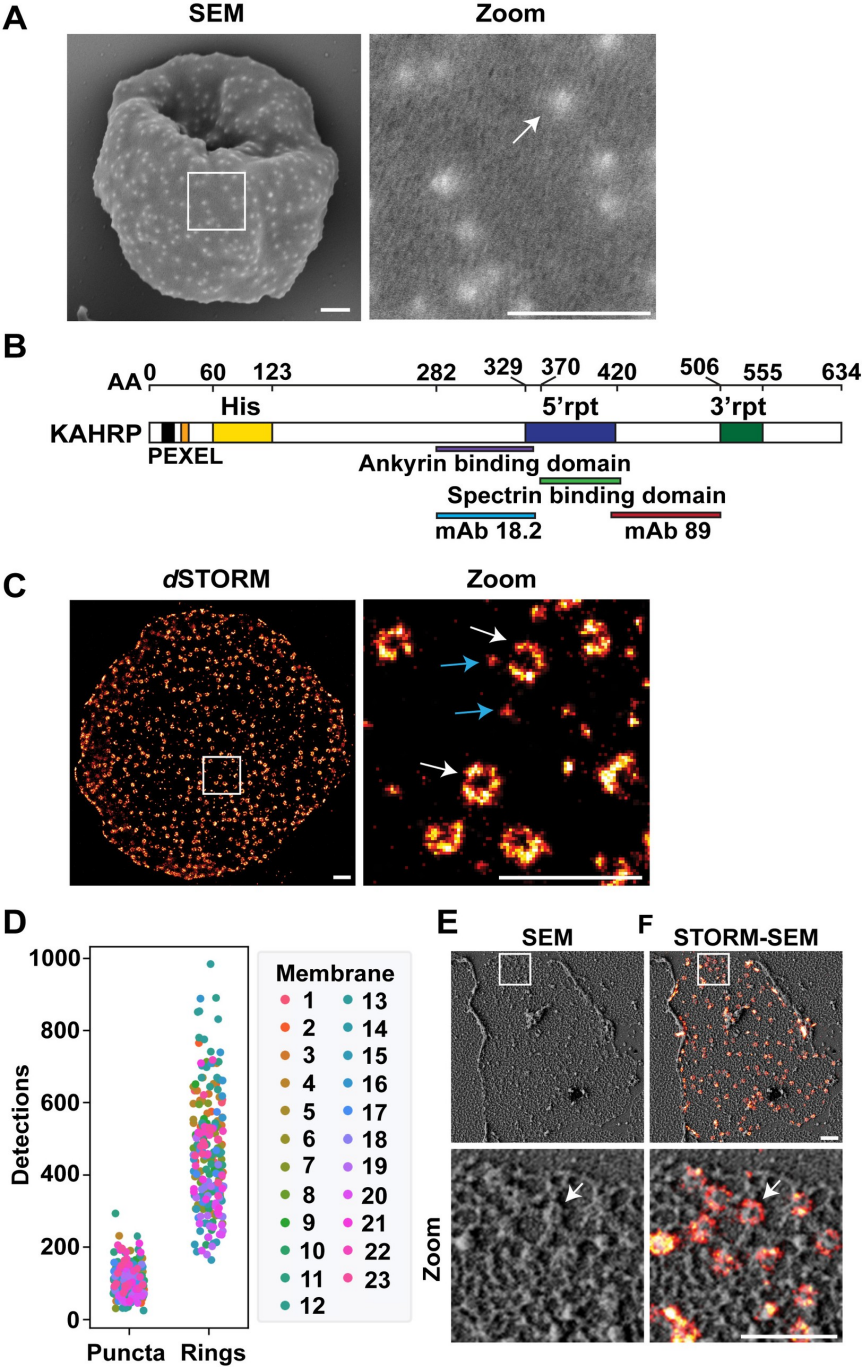
Visualizing knob structures at the external and internal surfaces. (A) SEM of an intact CS2-infected RBC (~36 h post-invasion). Zoom shows a representative 1 x 1 μm section. (B) Schematic representation of KAHRP. Protein domains and features are as follows: black–signal sequence; orange–PEXEL; yellow–histidine-rich (His) repeats (amino acids 60–123), blue—5’ repeats (amino acids 329–420), green—3’ repeats (amino acids 506–555), ankyrin-binding domain (amino acids 282–361), spectrin-binding domain (amino acids 370–441) and the binding regions of anti-KAHRP (mAb89: amino acids 424–539 and mAb18.2: amino acids 282–362). Full length CS2 KAHRP has 634 amino acids. (C) *d*STORM imaging of a sheared membrane preparation from CS2-infected RBCs (~30 h post-invasion) labelled with anti-KAHRP (mAb89) and anti-mouse Alexa-647 secondary antibody. Zoom shows a representative 1 x 1 μm section. Blue arrows: puncta. White arrows: ring structures. (D) The number of fluorescence events detected by *d*STORM within 200 x 200 nm regions drawn around individual anti-KAHRP labelled puncta and ring structures. Counts are presented from 23 (color-coded) sheared membranes. (E) Sheared membranes prepared from CS2-infected RBC membranes at 30 h post-invasion imaged by SEM. Zoom shows a representative 1 x 1 μm section. White arrow: Dimpled disk-structure. (F) STORM-SEM showing overlay of physical dimpled disks and anti-KAHRP labelled ring structures (white arrow). Scale bars: (A,C, E, F) 500 nm.

SEM imaging of the external surface of RBCs infected with CS2 strain *P*. *falciparum* (~30 h post-invasion) reveals knob structures as surface elevations (Fig 1A, zoom, white arrow). In agreement with previous reports the knobs have a diameter of ~90 nm [14]. While KAHRP is known to be a major component of the knob, its location within the structure and its mode of assembly at the RBC membrane skeleton are not clear.

In an effort to examine the physical organization of the knobs and their interaction with the host RBC membrane skeleton, we developed a correlative imaging method that allows analysis of the cytoplasmic surface of the RBC membrane. The RBC binding lectin, phytohemagglutinin, was cross-linked to glass coverslips and *P*. *falciparum*-infected RBCs (CS2 line, ~90% parasitemia, ~30 h post-invasion) were adhered tightly to the lectin[15]. A stream of hypotonic buffer was applied to remove the bulk of the cell, leaving a disc of remnant RBC membrane bound to the coverslip (S1A Fig).

Labelling of the remnant discs with a monoclonal anti-KAHRP antibody (mAb89) that recognizes an epitope in the C-terminal region of KAHRP [16] (Fig 1B) gives a diffuse immunofluorescence pattern in widefield deconvolution microscopy (S1B Fig), with individual knobs below the limit of resolution (~250 nm resolution in *xy*). This confirms that proteins that are tightly associated with the RBC membrane skeleton are retained after shearing (although it should be noted that weakly associating proteins could be lost).

We implemented the super-resolution microscopy technique, *d*STORM, which offers an *xy* resolution limit of about 20 nm [17]. Sheared membrane samples labelled with anti-KAHRP and anti-mouse Alexa Fluor 647-conjugated secondary antibody were mounted in a reducing and O_2_ scavenging buffer and imaged using Total Internal Reflection Fluorescence (TIRF) optics. Excitation with a 642 nm laser induces transfer of most of the Alexa-647 molecules to the triplet (dark) state, with only a subset of fluorophores emitting photons at any given time. Following collection of ~10,000 frames, a population of horseshoe or ring-shaped fluorescently-labelled KAHRP structures were observed (Fig 1C, zoom, white arrows).

The average distribution of the ring structures is very similar to that of the knobs when viewed from the external surface. The ring structures exhibit an external diameter (half maximum intensity) of 134 ± 3 nm and an internal diameter of 69 ± 4 nm (S1C and S1D Fig). Given the reported diameter of IgG (7–10 nm; [18]) and the secondary labelling strategy used in this work, the diameter of the KAHRP ring structure is consistent with the observed physical size of the knobs. The paucity of labelling in the center of the structure suggests a ring of assembled KAHRP modules.

### KAHRP puncta assemble into ring structures comprising discrete KAHRP modules

In addition to the ring-shaped structures, small puncta are observed with an apparent average diameter of 30 ± 2 nm (Fig 1C, zoom, blue arrows; S1C and S1D Fig). This size is consistent with the labeling of KAHRP with a primary and secondary IgG adding 7–10 nm per antibody. The diameters of the puncta are similar to the mean width of the ring walls (32 ± 1 nm), consistent with the ring structures being comprised of several individual KAHRP puncta (S1C and S1D Fig). Moreover, visual examination reveals 4 to 6 punctate components within many of the ring structures (Fig 1C, white arrow; S1C and S1E Fig). Where individual fluorescently labelled KAHRP elements were resolved, the average distance (mean ± S.D.) between the centers of the puncta within the rings was estimated to be 53 ± 10 nm (233 measurements from 18 membranes) (S1E Fig shows representative images).

We used the number of single molecule localization events (*i*.*e*. fluorescence emissions with sufficient photons for detection as individual events [19]) as a surrogate measure of the number of fluorescent molecules in different structures. Analysis of the raw data showed that puncta are associated with 102 ± 42 events (mean ± S.D) with a shoulder of higher values (S1F Fig), consistent with a dominant single species with a weak tendency to self-associate. The ring structures are associated with a mean of 459 ± 153 events (Fig 1D, S1G Fig), which equates to a mean ratio of 4.7 ± 2.1 puncta equivalents per ring structure (S1E Fig shows representative images). While further work is needed to confirm the structural basis for the fluorescence profile, the data suggest that the ring structures are comprised on average of five modular KAHRP units.

Following *d*STORM imaging, the sheared membranes were fixed, dehydrated, gold-coated, and then imaged using high-resolution SEM. The spectrin-actin network appears as bright (raised) skeletal elements over dark patches of background (Fig 1E). Close analysis of the SEM images reveals raised, dimpled disk-shaped structures that are closely integrated into the skeletal meshwork (Fig 1E, zoom, arrow). These dimpled disks have an average diameter of 85 ± 2 nm and a spacing and distribution (~8 per square micron) similar to that observed for knobs viewed from the external surface by SEM (Fig 1A, S1D Fig).

The *d*STORM and SEM data sets were aligned using landmark registration (see Methods) to produce a STORM-SEM image (Fig 1F). The KAHRP fluorescence signal lies around the raised disk-shaped structures, consistent with previous immuno-EM data showing KAHRP around the knob core [9] (Fig 1F, zoom, arrow). The flanking location of the *d*STORM ring structure around the periphery of the raised disk-shaped SEM structures is consistent with the additional width arising from the primary and secondary antibodies.

### Knob assembly occurs over the period of 16 h to 30 h post-invasion

**Fig 2:**
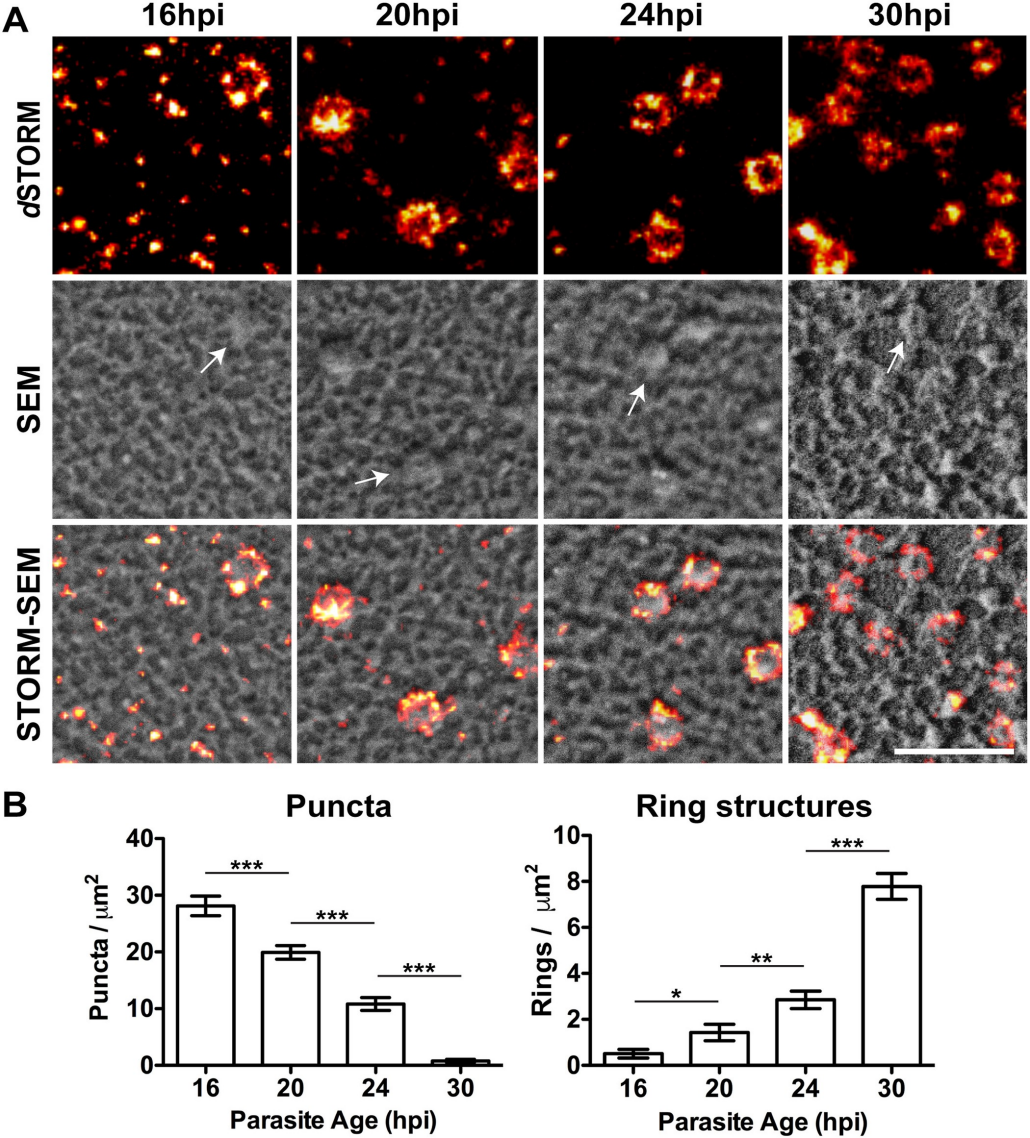
The density of KAHRP-labelled ring structures increases during parasite development. (A) CS2-infected RBC membranes imaged by *d*STORM and SEM during knob formation. Representative 1 x 1 μm sections are presented from cells at 16, 20, 24 and 30 h post-invasion (hpi). Scale bar: 500 nm. (B) The numbers of puncta (structures 5–50 pixels in size) and ring structures were quantified for multiple regions from different cells (n = 35, 25, 26 and 15 membranes for 16, 20, 24 and 30 h post-invasion respectively). Data is presented as the mean ± SEM (unpaired t-test, * p < 0.05, ** p < 0.005, *** p < 0.0001).

SEM-based imaging of the knobs at the external surface of CS2-infected RBCs reveals an increase in density from 1.3 to 9.4 per μm^2^ over the period 16–30 h post-invasion (S2A and S2B Fig). This timeframe is consistent with a previous analysis of knob formation [20], and with the reported timeframe for the delivery of PfEMP1 to the RBC surface [21]. We analyzed the organization of KAHRP-containing structures over this same period in sheared membranes. At 16 h post-invasion, a high density of puncta is evident with relatively few ring structures (Fig 2). During parasite maturation, the number of puncta decreases while the number of rings increases, eventually reaching 7.8 ± 0.6 per μm^2^, which is roughly equal to the number of knob structures observed from the external surface (9.4 ± 0.6 per μm^2^; S2A and S2B Fig). We note that the curved surface of whole infected RBCs may lead to a moderate overestimation of knob density at the external surface. Taken together, these data indicate that KAHRP is delivered to the membrane as modular units that are then assembled into the larger ring structures.

### Knob formation requires remodeling of RBC membrane skeleton actin

Maturation of infected RBCs has previously been reported to involve remodeling of the RBC membrane skeleton and redistribution of a population of actin molecules from the membrane skeleton into filaments that connect to parasite-derived structures called Maurer’s clefts [22, 23]. The F-actin depolymerizing drug, cytochalasin D, was previously shown to destabilize Maurer’s cleft-associated actin filaments in trophozoite-infected RBCs [22]. However, the effect of cytochalasin D on the rheological properties of infected RBCs and on the assembly of knobs has not been studied.

We examined the rheological properties of control and cytochalasin D-treated infected RBCs by assessing their ability to passage through a bed of microbeads designed to mimic the fenestrations in the splenic sinuses [24]. Tightly synchronized parasites (2 h window) were treated with cytochalasin D (10 μM) for 2 h prior to filtration at different time points across the ring to trophozoite transition (S3A Fig). The most dramatic effect was observed at 24 h post-invasion (S3A Fig). The lack of effect when infected RBCs were treated at 30 h post-invasion indicates that cytochalasin D can prevent but not reverse cellular rigidification.

**Fig 3:**
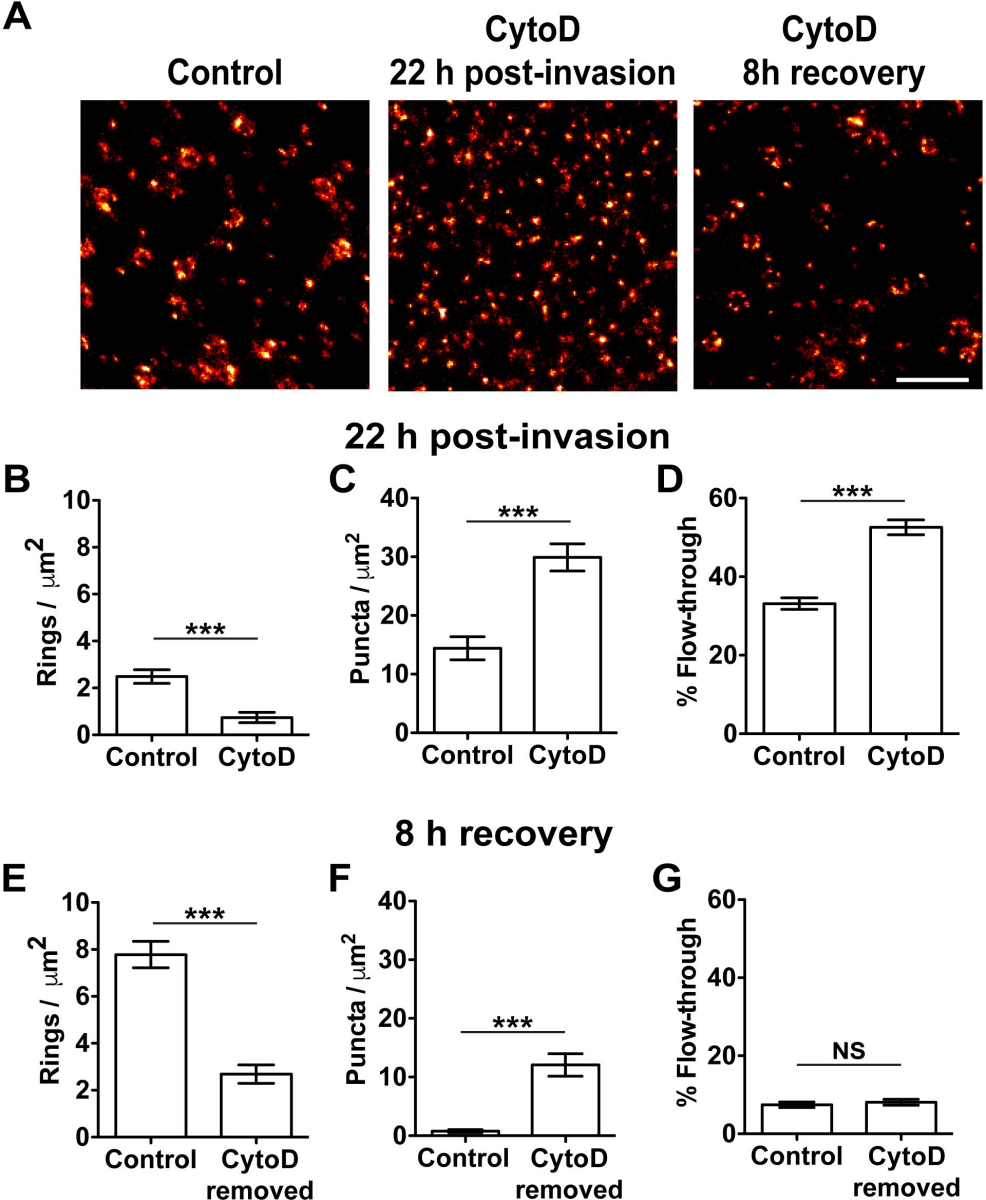
Actin remodeling is required for knob formation. Sheared membranes were prepared from CS2-infected RBCs treated with or without cytochalasin D (CytoD), from 16 h post-invasion. Parasite infected RBCs were harvested at 22 h post-invasion, or cytochalasin D was removed at 22 h and cells harvested 8 h after removal at 30 h post-invasion (CytoD 8 h recovery). (A) *d*STORM images showing KAHRP distribution. Scale bar: 500 nm. (B,C) Quantification of ring structures and puncta from untreated control (n = 31) and cytochalasin D-treated (n = 28) membranes at 22 h post-invasion (unpaired t-test, *** p < 0.0001). (D) Infected RBCs were passaged though a bed of microbeads and the parasitemia in the flow-through was assessed. Data represent three separate experiments each in triplicate (unpaired t-test, *** p < 0.0001). (E,F) Quantification of ring structures and puncta from control (n = 15) and cytochalasin D-treated (n = 30) cells following 8 h recovery (unpaired t-test, *** p < 0.0001). (G) Microbead filtration of cells following 8 h recovery showing no significant difference. Data represent three separate experiments each in triplicate. (Unpaired t-test, NS p = 0.53). Data is presented as the mean ± SEM.

We next examined the effect of treatment with cytochalasin D (10 μM) from 16 to 22 h post-invasion. While control infected RBCs assemble 2.5 ± 0.3 ring structures per μm^2^ by 22 h post-invasion, cytochalasin D-treated infected RBCs exhibit only 0.7 ± 0.2 ring structures per μm^2^ (Fig 3A and 3B). Accordingly, the cytochalasin D-treated cells exhibit a higher density of small KAHRP puncta, showing that cytochalasin D treatment does not affect KAHRP delivery and binding to the RBC membrane (Fig 3B and 3C). STORM-SEM imaging confirms the loss of the disk-shaped physical knob structures (S3B Fig). Associated with this loss of physical knob structures, cytochalasin D-treated infected RBCs show increased ability to passage through the filter (Fig 3D).

To determine whether the effects of cytochalasin D are reversible, treated cells were washed and allowed to recover for 8 h (*i*.*e*. re-examined at 30 h post-invasion). *d*STORM imaging reveals an increase in the number of ring structures (2.7 ± 0.4 per μm^2^), although the density does not reach the level of the controls (Fig 3E). The number of puncta remains higher than in the control (Fig 3F). Despite the lack of ring structures, the treated cells show very low filterability following recovery, similar to that of controls at 30 h post-invasion (Fig 3G).

To confirm that the cytochalasin D-mediated effects are not due to a loss of parasite viability or to developmental delay, we measured parasite area (in Giemsa-stained smears). Incubation with cytochalasin D over the 16–22 h post-invasion period had no effect on the parasite size (S4A and S4B Fig). Similarly, upon removal of the cytochalasin D, the parasites were able to progress to the next cycle (S4C Fig). By contrast, extending the cytochalasin D incubation (16–30 h post-invasion) ablated parasite viability (S4C Fig), preventing examination of the effects of a longer period of treatment on parasite physical properties.

### Dissection of the regions of KAHRP needed for skeleton binding and ring complex assembly

The N-terminal region of KAHRP contains a signal sequence, a PEXEL motif (needed for export) and a histidine-rich region (Fig 1B) [25, 26]. The C-terminal half of KAHRP includes two repetitive sequence elements (the so-called 5´ and 3´repeats; Fig 1B) that have been reported to be involved in binding to the membrane skeleton [12, 27]. Recent work suggests that the 5’ repeat region plays the most important role in binding to spectrin [10].

Building on a previous study [12], we generated transfectants expressing KAHRP truncated at amino acid 362 (K362), 405 (K405) or 530 (K530), in CS2 parasites, by 3’ replacement of the *kahrp* locus, using the selection-linked integration approach (S5A Fig) [28]. Expression of truncated protein was confirmed by Western blotting (S5B Fig), using mAb18.2 which binds an epitope in the N-terminal region of KAHRP. Immunofluorescence microscopy of infected RBCs at ~35–40 h post-invasion reveals non-homogeneous labelling at the host RBC membrane for full-length KAHRP and the K530 line (which retains the spectrin-binding domain). In contrast, the K405 line (which has a partly truncated spectrin-binding domain) and the K362 (which lacks most of the high-affinity spectrin-binding domain) show more diffuse immunofluorescence signals (S5C Fig) with lower intensity (S5D Fig). SEM imaging of whole infected RBCs reveals surface protrusions on the K530 which closely resemble those of wildtype CS2 (S6A and S6B Fig, arrows), consistent with the previous data for these truncations in 3D7 [12]. The K405 line exhibits fewer protrusions on the external surface (S6A and S6B Fig), while the K362 line lacks classical knobs (S6A and S6B Fig).

**Fig 4:**
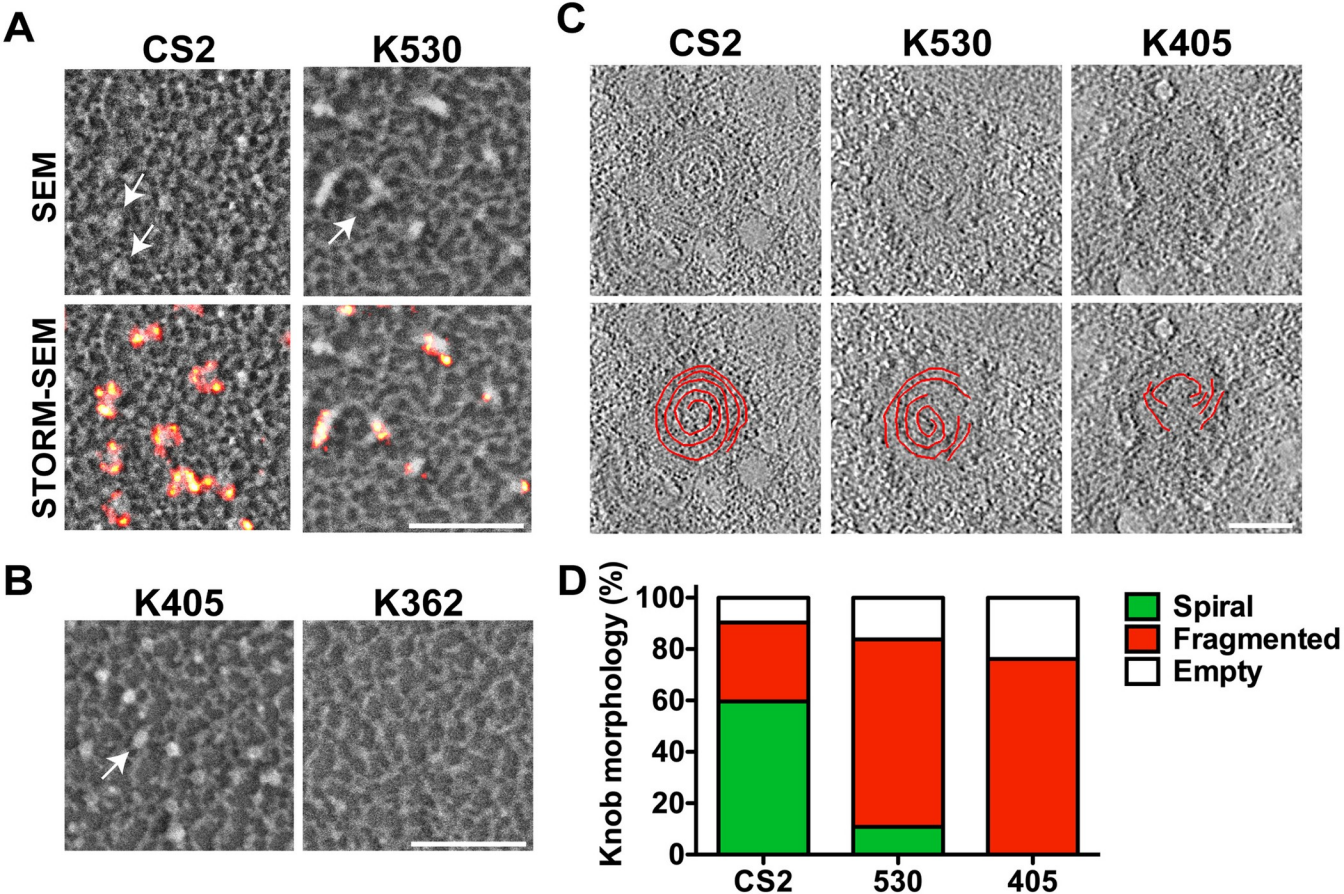
Spectrin binding is required for correct knob formation. (A,B) Sheared membranes prepared from RBCs infected with CS2, K530, K405 and K362 parasite lines at 40 h post-invasion were imaged by SEM and *d*STORM (A; anti-KAHRP mAb18.2) or only SEM (B). Images are displayed as 1 x 1 μm zoomed panels. Scale bar: 500 nm. (C) Projections from electron tomograms of negatively-stained preparations of schizont-infected RBCs from the CS2, K530 and K405 lines. The spiral structures were segmented manually, shown as an overlays in the bottom panel. Scale bar: 50 nm. (D) The proportions of knobs containing complete, fragmented or no spiral were recorded (n = 52, 24 and 21 knobs for CS2, K530 and K405 infected RBCs, respectively). Data is presented as a percentage of the total number of recorded knobs.

SEM of sheared membranes prepared from infected RBCs at 40 h post-invasion reveals raised structures in the wildtype parasites and the K530 and K405 mutants (Fig 4A and 4B, arrows), consistent with the presence of protrusions at the external surface (S6A and S6B Fig), but not in the K362 mutant (Fig 4B and S6A and S6B Fig). While the wildtype parasites exhibit characteristic dimpled, disk-shaped structures, the K530 and K405 mutants exhibit elongated or rounded bauble-like structures (Fig 4A and 4B, arrows). *d*STORM imaging of anti-KAHRP-labelled membranes reveals ring structures in wildtype CS2 parasites (30 h post-invasion; 8 ± 1 per μm^2^) (Fig 4A, S6C Fig). In the K530 mutant, the fluorescence signal is located at the periphery of the elongated baubles (Fig 4A, bottom right). The total number of fluorescent features is lower in the K530 mutant (5 ± 0.3 per μm^2^), reflecting the reduced number of knobs visible on intact cells (S6C and S6D Fig). Interestingly the number of puncta in the K530 parasites is elevated compared to wildtype (1.5 ± 0.2 per μm^2^) (S6C Fig). Even in schizont stage parasites (40 h post-invasion) the K530 line exhibits fewer ring structures than wildtype parasites, but similar numbers of puncta (S7A and S7B Fig). No KAHRP protein is detected by *d*STORM imaging of sheared membranes prepared from K405 truncation mutants, despite the presence of physical structures (Fig 4B, left). The lack of fluorescence signal (either puncta or ring structures) may indicate loss of KAHRP during preparation of the sheared membranes. The K362 mutants exhibit neither physical knobs, nor evidence for the presence of KAHRP.

We used electron tomography to examine the aberrant knob structures–a method that has previously been shown to reveal a spiral structure within the knobs (9). While complete preservation of the supporting structure is not achieved in negatively-stained membrane preparations, we observed well-preserved spirals in 59% of the knobs (electron-dense structures) examined in wildtype CS2 parasites (Fig 4C and 4D, S8 and S9 Figs). In contrast, knob-like structures from the K530 mutants show complete spiral structures in only 11% of the knobs examined, with the majority (73%) showing fragmented and discontinuous spiral structures (Fig 4C and 4D, S8 and S9 Figs). The observed degradation of the supporting structure is consistent with the malformed knobs, as viewed by SEM of the external surface (S6 Fig). No complete spirals are observed in the K405 mutants, but 76% of the knobs exhibit fragmented spiral structures (Fig 4C and 4D, S8 and S9 Figs). We also observed electron-dense structures that did not contain spirals (empty knobs), particularly in the truncation mutants (CS2, 10%; K530, 16% and K405, 24%) (Fig 4C and 4D, S8 and S9 Figs). We also note that the maximum diameter of the spirals in the K530 (47 ± 3 nm) and K405 mutants (39 ± 2 nm) is smaller than in wildtype CS2 (54 ± 4 nm) parasites (S9B Fig). Similarly, the mean combined length of the spiral features (both fragmented and whole) in individual knobs is shorter in the K530 (233 ± 25 nm) and K405 (156 ± 12 nm) mutants than in wildtype parasites (322 ± 40 nm) (S9C Fig). The electron-dense knob coat has a slightly decreased diameter in the K530 (87 ± 3 nm) and K405 (80 ± 4 nm) mutants when compared to CS2 (89 ± 3 nm), consistent with our analysis of SEM images of the external surface (S9D, S6A and S6B Figs).

### PfEMP1 is delivered to the membrane and targeted to the knobs

The mechanism for trafficking of PfEMP1 to knobs is poorly understood, in part due to a lack of antibody reagents that recognize PfEMP1 within the context of the knob structure [29]. In this work, we made use of transfected parasites (A4 background) expressing a mini-PfEMP1-GFP construct, A4var-V5-TM-ATS-GFP (referred to here as PfEMP1A-GFP) [30, 31]. This minimal PfEMP1 contains the semi-conserved N-terminal region (DBL and CIDR), the transmembrane segment and the C-terminal ATS domain of the A4 *var* allele. It was previously shown that PfEMP1A-GFP molecules are delivered to the RBC surface [31].

**Fig 5:**
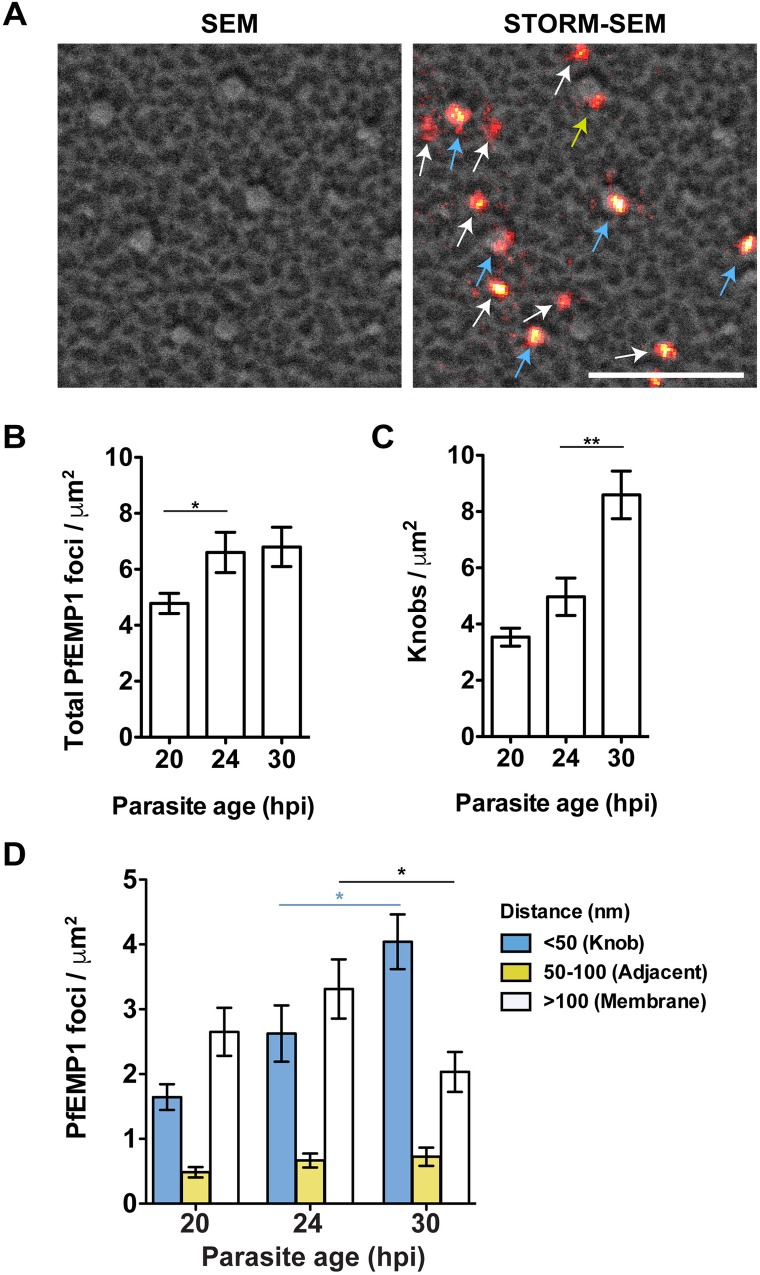
PfEMP1 associates with knobs laterally. (A) Sheared membranes prepared from RBCs infected with PfEMP1A-GFP transfectants at 20 h post-invasion. Samples were imaged by SEM and *d*STORM (anti-GFP). STORM-SEM shows fluorescent foci at knobs (blue arrows), adjacent to knobs (yellow arrows) or independent from knobs (white arrows). Scale bar: 500 nm. (B) Total number of PfEMP1A-GFP foci on sheared membranes at 20, 24 and 30 h post-invasion. (C) Knob counts from sheared membranes viewed by SEM. (D) Quantitation of the number of fluorescent anti-GFP foci and SEM knobs per μm^2^ from RBC membranes infected with A4 PfEMP1A-GFP parasites at 20, 24 and 30 h post-invasion (n = 11, 12 and 10 membranes, respectively). Data plotted as fluorescent foci at a knob (<50 nm, blue), adjacent to a knob (50–100 nm, yellow) or independent from knobs (>100 nm, white). See S10B Fig for explanatory diagram.

We prepared sheared membranes from PfEMP1A-GFP transfectants and detected the transgene product using anti-GFP and anti-mouse Alexa Fluor-647 antibodies (Fig 5A, S10A Fig). Bright fluorescent PfEMP1 foci were observed with a signal intensity suitable for *d*STORM imaging. By contrast, antibodies directed against the acidic terminal sequence (ATS) domain of endogenous PfEMP1 did not provide sufficient signal for *d*STORM imaging, suggesting that the antibody does not bind with high enough affinity to membrane-inserted PfEMP1 to permit single molecule localization microscopy. STORM-SEM imaging of PfEMP1A-GFP sheared membranes was performed at 20, 24 and 30 h post-invasion and PfEMP1 foci and knob structures counted (Fig 5B and 5C). The number of PfEMP1A-GFP foci at the membranes increases from 20 to 24 h post-invasion, then remains constant through to 30 h post-invasion (Fig 5B). The numbers of knobs at different stages of development in the PfEMP1A-GFP parasites is similar to that seen for wildtype CS2 parasites (Fig 5C and S2 Fig). Examination of the STORM-SEM images reveals three different populations of PfEMP1A-GFP, which we have classified as being at the knobs (blue arrows, within 50 nm from a physical knob center), adjacent to the knob (yellow arrows, within 50–100 nm from a knob center) and greater than 100 nm from the center of a knob (white arrows, Fig 5A, S10B Fig). We applied this criterion and analyzed the sheared membranes to determine the location of PfEMP1A-GFP at time points over the period of virulence complex assembly. At 20 h post-invasion only 34% of the PfEMP1A-GFP foci are located at knobs, increasing to 40% at 24 h post-invasion, and reaching 59% at 30 h post-invasion (Fig 5D). Our data are consistent with PfEMP1 being delivered to the RBC membrane in regions away from knobs and then moving laterally to associate with and be assembled into knob structures. Alternatively, it is possible that knobs disassemble and reassemble around PfEMP1 foci.

## Discussion

We developed and implemented a multimodal imaging technique that combines *d*STORM localization microscopy and SEM. The technique allows us to map the locations of KAHRP and PfEMP1 to physical structures assembled at the RBC membrane skeleton. We show that KAHRP is delivered as punctate structures (with a diameter close to the resolution limit, *i*.*e*. ~30 nm) that associate with the spectrin meshwork, from about 16 h post-invasion. As the parasite matures, larger (~130 nm diameter) ring-shaped structures appear and increase in number, while the puncta disappear.

The quantification of signal intensities from *d*STORM data is challenging, particularly in the context of indirect labelling with antibodies [32]. Nonetheless, analysis of localization events for the individual fluorescent puncta reveals a narrow distribution of fluorescence intensities, suggesting a dominant single species, consistent with a modular population, potentially comprising single KAHRP proteins, KAHRP containing complexes or very tightly self-associated KAHRP oligomers.

**Fig 6:**
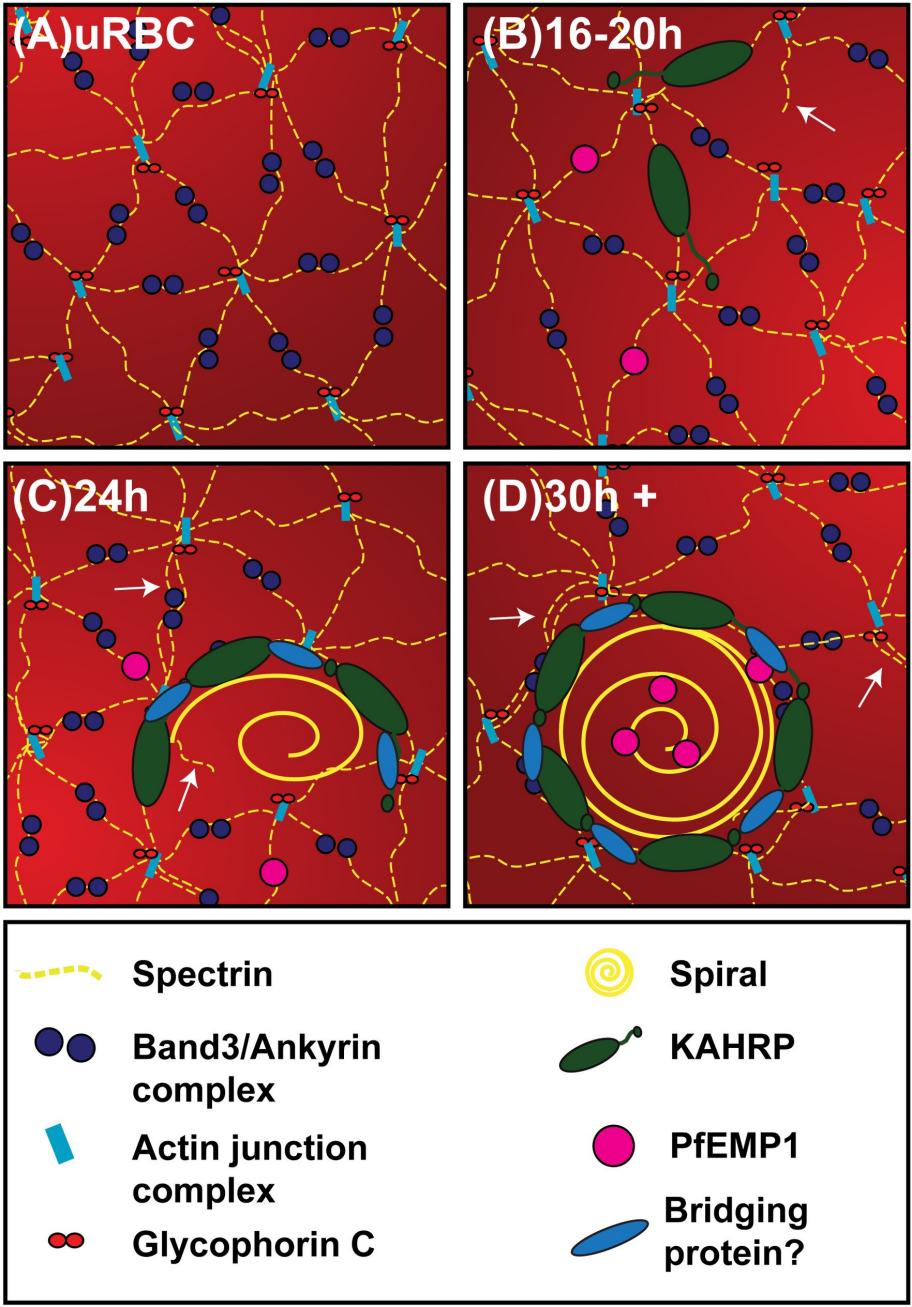
A model for virulence complex formation. A schematic diagram of the proposed model for assembly of the virulence complex. (A) The membrane skeleton of an uninfected RBC (uRBC). (B) KAHRP is delivered to the RBC membrane skeleton where it interacts with spectrin. Host cell remodeling is initiated (white arrows) via the dissociation of the actin junction points, allowing spectrin remodeling. PfEMP1 is delivered to the RBC membrane. (C) Host cell remodeling continues with spectrin octamers being formed (white arrow). KAHRP undergoes higher order assembly (with potential involvement of a bridging protein) forming a horseshoe-shaped structure that scaffolds the spiral knob core. PfEMP1 moves laterally to associate with the virulence complex. (D) KAHRP modules form the base of the knob complex. Most of the PfEMP1 is assembled adjacent to or at knob complexes.

Our analysis of the number of localization events suggests that the ring structures comprise an average of 5 KAHRP modules, each separated by an average distance of ~50 nm. Recent work showed that the KAHRP 5’ repeats form an extended interaction interface with domains 10–14 of β-spectrin, which lie near to the ankyrin-binding domain [10]. One explanation for the 5-module ring structure is that the ring is underpinned by 5 spectrin tetramers, each presenting a KAHRP binding site (separated by ~50 nm). This suggestion is consistent with recent estimates of the average branch length of the spectrin network in trophozoite stage-infected RBCs, *i*.*e*. 49–64 nm [15, 23, 33] and with a study showing that an average of 5 spectrin proteins converge per junctional complex in the RBC membrane skeleton [34]. Given its unusual amino acid composition and predicted disordered domains [10], we also considered the possibility that KAHRP adopts a highly elongated shape. Propagation of a weak self-association reaction could permit the formation of a five-membered ring, with individual elements separated by ~50 nm (see Fig 6). Another possibility is that an as yet unidentified bridging protein facilitates the assembly process. It is also possible that the shearing method strips loosely associated structures and proteins (including KAHRP) from the membrane. The proposal that only about 5 KAHRP molecules contribute to the knob structure means that KAHRP is much sparser than envisaged in previous models (*e*.*g*. see [10]) and further work is needed to test this model.

SEM of the sheared membranes reveals dimpled, disk-shaped structures (~85 nm diameter) associated with the membrane skeleton. Correlative imaging shows that these disks sit neatly inside the ring of anti-KAHRP fluorescence. We suggest that the dimpled disks represent the physical base of the knob structures. A recent ultrastructural study visualized the knob complex as a spiral structure connected by multiple links to the RBC membrane skeleton and coated by an electron dense layer that is likely composed of KAHRP molecules [9]. In this work, we used high resolution images of negatively-stained membranes to confirm the presence of spiral structures, with a diameter of 54 nm surrounded by an electron dense coat with a diameter of 89 nm (for CS2 parasites). While it is possible that the knob structure may be altered during the preparation of samples for negative staining and EM, our data are consistent with KAHRP molecules surrounding a spiral scaffold that underpins the knob complex. Of interest, our data show that there is a region in the center of the knob complex that does not contain KAHRP, which may represent the region where PfEMP1 is displayed. However, it does not discount the possibility that other modules of KAHRP may be present (and not antibody accessible) or that they may have been lost in the shearing process.

The membrane skeleton of the RBC is reorganized during intraerythrocytic development of *P*. *falciparum* [15, 35], potentially as a result of the mining of actin for use in filaments that connect to the Maurer’s clefts [22]. Cytochalasin D, a cell-permeable mycotoxin that binds to the slow-growing ends of actin filaments with high affinity [36], has been shown to block RBC membrane skeleton remodeling [22, 23]. We found that cytochalasin D treatment blocks knob formation. Treatment does not prevent binding of KAHRP nor affect the timing of KAHRP delivery to the RBC membrane skeleton but inhibits the reorganization of KAHRP modules into ring structures, and prevents the physical assembly of knobs. We show that the block is not due to a developmental arrest or a loss of parasite viability. This data strongly suggests that correct actin organization and/or skeletal reorganization are needed to support correct assembly and anchoring of the knob. Alternatively, it is possible that cytochalasin D treatment inhibits the trafficking of unknown third party proteins that are required for knob assembly or that cytochalasin D treatment impairs knob anchoring.

The assembly of knobs stiffens the spectrin network [24, 37], through composite strengthening, strain hardening, and knob density-dependent vertical-coupling mechanisms [38]. Similarly, the reorganization of the skeletal network, with an increased number of stretched spectrin tetramers and octamers converging on a reduced number of junction points, is predicted to increase the shear modulus of the membrane skeleton [38]. Cytochalasin D-treated infected RBCs show enhanced filterability through a microbead bed compared with control cells, consistent with important roles of knob assembly and skeleton reorganization in rigidifying the host RBC membrane. Upon removal of cytochalasin D, partial recovery of knob formation is observed, and the infected RBCs no longer survive filtration, indicating restoration of cellular rigidity.

Using C-terminal truncations of KAHRP (based on previous work [12]) we investigated the role of sequence information within the C-terminal region of KAHRP in driving formation of the ring structure. We found that transfectants expressing KAHRP truncated at residue 530 exhibit malformed (elongated bauble-like) physical structures. KAHRP modules are associated with these malformed knobs, but they exhibit fewer (and more fragmented) spiral structures with a smaller diameter and a reduced length. This indicates that the information for KAHRP self-association is retained in the K530 truncation but that the sequence information in the 3’ repeat and C-terminal sequence is required for formation of canonical ring structures.

The 5’ repeat region of KAHRP has been shown to be the major contributor to spectrin binding [10, 12]. When KAHRP is truncated at residue 405 (mid-way through the 5’ repeats), malformed knob-like protrusions are still observed by external SEM, and bauble-like structures are observed at the internal surface. Some anti-KAHRP signal is observed (by immunofluorescence microscopy of whole cells) in K405 mutant-infected RBCs, but no anti-KAHRP signal is observed in sheared membrane samples, prepared for *d*STORM imaging. This suggests that the K405-KAHRP may be free in the cytoplasm or only weakly associated with the RBC membrane. In negatively-stained preparations of the K405-infected RBCs, no well-preserved spiral structures are observed, and the degraded and collapsed spiral structures exhibit significantly reduced diameter and length. Truncation of KAHRP at residue 362 completely ablates knob formation. These data confirm that the 5’ repeat region of KAHRP is needed for binding to spectrin. It further demonstrates that KAHRP binding to spectrin is needed for the formation and stabilization of the spiral structure but that KAHRP, itself does not appear to be a physical component of the spiral. Whether the spiral protein comprises another parasite-derived protein or reorganized RBC skeletal proteins remains to be determined. Collectively this data suggests a model where the C-terminal region of KAHRP is needed for the correct organization of KAHRP into rings structures, the correct assembly of the spiral structure and the acquisition of canonical knob morphology (S11 Fig).

While PfEMP1 can be surface-displayed in the absence of knobs [39] and knobs can be assembled in parasites with a PfEMP1 trafficking defect [12, 40], recent work suggests an interplay between the PfEMP1 variant that is expressed and the density of knobs at the RBC membrane [41]; and an early study suggested that the virulence complex might be pre-assembled at the Maurer’s clefts [42]. Similarly, PfEMP1 variants show different affinities for spectrin and different members of the PHIST gene family [10, 43]. Thus, the precise mechanism for assembly of the virulence complex remains unclear. In this work we observed three sub-populations of PfEMP1 at the RBC membrane, located either directly at, adjacent to, or at some distance from knobs. The percentage of knob-associated PfEMP1 foci increases over the period of virulence complex assembly (20 h– 30 h post-invasion). While caution should be exercised in extrapolating native PfEMP1 trafficking from the behavior of a tagged PfEMP1 construct, the data are consistent with gradual assembly of PfEMP1 into preformed knobs by lateral transfer.

Taken together with previous work, our data point towards a model of virulence complex assembly that can be interrogated with further experiments. We propose that the individual virulence complex components are assembled *in situ* at the RBC membrane skeleton rather than being delivered as a pre-assembled complex (Fig 6A–6D). We suggest that KAHRP molecules are trafficked to the RBC membrane from ~16–20 h post-invasion, with the spectrin-binding domain driving association with the membrane skeleton (Fig 6B). We propose that reorganization of the spectrin-actin network, driven by loss of actin junction points, allows higher order assembly of KAHRP molecules that are bound to adjacent spectrin tetramers (potentially facilitated by a bridging protein, such as the spiral protein), leading to the assembly of rings of KAHRP modules (Fig 6C and 6D). We propose that individual KAHRP modules move laterally across the membrane skeleton prior to assembly. Alternatively, modules that fail to assemble may disassociate, leaving only the successfully built rings. We propose that the spiral scaffold is then assembled on the KAHRP ring in the region of destabilized membrane skeleton (Fig 6D). We propose that PfEMP1 is delivered to and inserted into the RBC membrane at random locations, where it interacts weakly with spectrin. We suggest that PfEMP1 is then transferred laterally into the central region of the preformed knob structures. Further experiments are needed to test various elements of this model.

Assembly of PfEMP1 into raised knob platforms likely promotes adhesion by presenting the receptor-binding domains of PfEMP1 above the RBC glycocalyx. The rigidification of the RBC membrane that results from knob assembly and skeletal reorganization also facilitates adhesion of infected RBCs by distributing the tensional forces imposed on individual receptor-bound PfEMP1 molecules across the entire knob region and through to the membrane skeleton [38]. The insights we have gained into the knob assembly process may lead to new ways to inhibit the presentation of PfEMP1 on infected RBCs, and thus new ways of interfering with this lethal pathogen.

## Materials and methods

### Ethics statement

Red Blood Cells and serum were obtained from the Australian Red Cross blood service. All blood products were anonymous and individual donors could not be identified. This work was approved by the University of Melbourne Human Research Ethics Committee (Approval number 1135799).

### Parasite culture

*P*. *falciparum* (wildtype CS2[44] and A4 expressing the A4var-V5-TM-ATS-GFP construct (PfEMP1-A) [31]) was cultured in O+ RBCs at 5% haematocrit in complete culture media containing RPMI-GlutaMAX-HEPES (Invitrogen) supplemented with 5% v/v human serum (Australian Red Cross blood service), 0.25% w/v AlbuMAX II (Invitrogen), 200 μM hypoxanthine, 10 mM D-glucose (Sigma) and 20 μg/ml gentamicin (Sigma). Parasites were synchronsied to ring stage with 5% D-sorbitol[45] or enriched at mature stage using Percoll purification or magnetic separation [46, 47]. Parasites were synchronsied to a 2 h post-invasion window as previously described [48]. Knob positive parasites were selected on gelatin [49]. Parasites were treated with 10 μM cytochalasin D as previously described [30]. Parasite survival assays were performed by diluting the cytochalasin D treated parasites by half by adding fresh RBCs and media to the culture following washout of the drug. The parasitemia was assessed by Giemsa smears in the next parasite cycle.

### Plasmid constructs and transfection

Parasite lines expressing C-terminal truncations of KAHRP at amino acid 362, 405 and 530 were created in the CS2 parasites, as previously described for 3D7 [12]. A 3’ replacement approach using the selection linked integration was used to create these truncations [28]. The targeting sequences for each of the truncations were PCR amplified from 3D7 genomic DNA using the common KtruncF (5’-gcggccgcTAAAATAATGGAAACGGATCCGGT-3’) and either the K362R (5’-gtcgacTTAGGAATGGTGTTTTTCATCACC-3’), K405R (5’-gtcgacTTATTTTACGCTTTCTGCATCTTC-3’) and K530R (5’-gtcgacAGTACTAATGCCGCAACAAATTAA-3’) primers. The resulting PCR products were then digested and directionally cloned into the NotI and SalI digested pSLI-TGD plasmid. This leads to the removal of the GFP from this plasmid. The constructs were then transfected as previously described and maintained on media supplemented with WR99210 (5 nM) [50]. Selection linked integration was performed as previously described [28].

### Membrane shearing

Coverslips were cleaned in acetone and 50% methanol, treated with 3-aminopropyl triethoxysilane (APTES), bis-sulfosuccimidyl suberate, and incubated with the ligand erythroagglutinating phytohemagglutinin (PHAE) [15]. Enriched infected RBCs were immobilized on the functionalized glass slides, then sheared by applying 5P8-10 buffer (5 mM Na2HPO4/NaH2PO4, 10 mM NaCl, pH 8) from a 30 mL syringe (23-G needle) at an angle of ~20° [23]. The membrane disks were placed in PBS prior to imaging.

### Scanning electron microscopy

Coverslips with adhered infected RBCs or sheared membranes were placed in a humid chamber and fixed with 2.5% glutaraldehyde in PBS for 1 h at room temperature before washing 3 times with PBS, and submerging in H_2_O for 5 min, then sequentially for 5 min each in 20%, 50%, 70%, 80%, 90%, 95% and 100% ethanol. Coverslips were dried using a compressed nitrogen gas nozzle and stored in a vacuum desiccator. Coverslips were gold-coated for 40 s at 25 mA using a Dynavac sputter coating instrument. The coating thickness was measured at 0.2 nm on a quartz crystal microbalance. Images were acquired using an FEI Teneo SEM using the ETD detector in Optiplan mode, at a working distance of 5 mm, a beam current of 50 pA and a 2-kV accelerating voltage.

### Immunofluorescence microscopy

Parasite samples for immunofluorescence assays were prepared either by fixation of thin blood smears with ice cold 90% acetone/ 10% methanol, or by immobilization of infected RBCs to PHAE ligand followed by fixation with 4% paraformaldehyde/0.0065% glutaraldehyde as previously described [14, 51]. All antibodies were diluted in 3% BSA/1XPBS for use. The following primary antibodies were used in this study; anti-KAHRP (mAb89, 1:500) [16], anti-KAHRP (mAb18.2, 1:500)(obtained from the European Malaria Reagent Repository), anti-GFP (mouse, 1:500, Cat. No. 11814460001, Roche). Primary antibodies were detected using Alexa Fluor 488 or 647 secondary antibodies (goat, 1:1,000, Cat. No. A11001, A11008, A21235, A21244, Invitrogen). Parasite nuclei were stained with DAPI. Primary and secondary antibody incubations were performed at room temperature for 1–2 h. The slides were washed 3 times in 1XPBS between antibody incubations. The slides were mounted in *p*-phenylenediamine antifade and kept at 4°C. Samples were imaged on a DeltaVision Elite Restorative Widefield Deconvolution Imaging System (GE Healthcare) using the 100x UPLS Apo (Olympus, 1.4NA) objective lens under oil immersion. Samples were excited with solid state illumination (Insight SSI, Lumencor). The following filter sets with excitation and emission wavelengths were used: DAPI Ex390/18, Em435/48; FITC, Ex475/28, Em523/26; TRITC, Ex542/27, Em594/45; Cy5 Ex 632/22, 676/34 nm. Images were processed using the FIJI ImageJ software [52]. Immunofluorescence quantification was performed using FIJI. The fluorescence intensity of the KAHRP and spectrin signals (minus background intensity) were measured. The KAHRP fluorescence relative to spectrin is plotted, a minimum of 10 cells were analysed for each group.

### *d*STORM immunofluorescence microscopy

Immunolabeled membrane samples were loaded into a Chamlide chamber and immersed in a buffer containing 10% w/v glucose, 50 mM Tris pH 8, 10 mM NaCl, 10 mM cysteamine hydrochloride, 40 μg/mL catalase, 0.56 μg/mL glucose oxidase. Samples were imaged using the DeltaVision OMX V4 Monet Localization Microscopy system (GE Healthcare), with 642 nm laser TIRF illumination (60X oil immersion lens, Olympus, 1.49 NA) and a beam concentrator to assist fluorophores transition to the triplet (dark) state. A 405 nm laser was used to improve fluorophore cycling back to the ground state. Single events were captured over 10,000 frames. DeltaVision softWoRx (GE Healthcare) software was used to process data. Fluorescent events with a localization precision of 5–100 nm and persistence between 1–100 frames were included in the final reconstruction. Each event was fitted with a Gaussian distribution to localize the point of origin. Data was recolored in FIJI using the ‘Red Hot’ look-up table, where black, red, yellow and white indicate a gradient from low to high signal.

### Electron tomography

TEM grids with Formvar support film were coated with carbon and glow discharged. The grids were functionalized with 0.1% poly-L-lysine solution for 5 min before incubation with 2% infected RBC cell suspension in PBS. Unattached cells were washed away from grids by dipping in PBS. The cells were lysed with hypotonic 5P8Q10 buffer (5 mM Na_2_HPO_4_/NaH_2_PO_4_, 10 mM NaCl, pH 8.0) for 1 min. Lysed cells were negatively stained with 1% aqueous uranyl acetate solution for 30 sec. Before imaging the back side of the grids were layered with 6 nm fiducial gold. Tilt series were collected with single tilt holder from -70° to 70° using an FEI Tecnai G2 F30 (FEI Company, Hillsboro, OR) electron microscope with a field emission gun operated at 200 kV. Tomograms were aligned and reconstructed using IMOD/Etomo (Boulder Laboratory for 3D Electron Microscopy of Cells). Samples from wildtype CS2 and the truncation mutants were prepared, stained and imaged in parallel on the same day.

### Immunoblotting

Wildtype CS2 and truncation mutant trophozoite stage parasites were enriched from culture by Percoll purification and subjected to lysis with 0.03% Saponin/1XPBS as previously described [53]. The pellet fraction from saponin lysis was mixed with LDS sample buffer (Invitrogen) and DTT (Invitrogen) and separated on 4–12% Bolt Bis-Tris gels (Invitrogen), using MOPS buffer. Gels were then transferred to 0.2 μm nitrocellulose membrane using the iBlot 2 system (Invitrogen). The membranes were blocked in 3.5% skim milk in PBS for at least 1 h at room temperature prior to addition of the primary antibody. Anti-KAHRP (mAb18.2, 1:500) primary antibody was diluted in 3.5% skim milk/PBS and incubated on the membranes overnight. Anti-mouse (goat, 1:25,000, Promega) horseradish peroxidase-conjugated secondary antibodies in 3.5% skim milk/PBS were incubated for 1 h at room temperature. Membranes were washed between antibody incubations in 1XPBS/0.05% Tween20. Membranes were incubated with Clarity ECL substrate (BioRad) and imaged using the ChemiDoc MP Imaging System (BioRad).

### Microbead filtration

The spleen mimic filtration method was used to assess the deformability properties of parasite infected RBC [24]. Briefly, synchronous parasites at the desired age were prepared at 6% parasitemia and 1% hematocrit in 1% AlbuMAX II in 1X PBS. The parasite material was flowed using a syringe pump over the microbead matrix at a flow rate of 60mL/min. A total of 8 mL of 1% AlbuMAX II/PBS was flowed through the bead matrix and the flow through collected and centrifuged to collect the parasites. The data presented is the percentage of parasites present in the flow through relative to starting parasitemia. Each experiment is performed in triplicate on at least 3 separate occasions, the mean and standard error are plotted, and a t-test is used to evaluate statistical significance.

### *d*STORM ring structure and puncta measurements

Ring structure measurements were performed on sheared membranes from parasite infected cells at 30 h post-invasion using FIJI. A line was drawn across the center of the puncta or ring structure and the profile plotted. The half maximum of each peak was extracted from the raw data and was used to generate the half maximal width of the structures. The puncta diameter and the outside and inside diameters of the rings were calculated. The width of the ring wall is also calculated. A total of 10 puncta or 5 individual rings from 5 separate membranes were analysed and the mean ± the standard error plotted.

### Counts of ring structures and puncta from *d*STORM data

Ring structures and puncta were counted from at least 2 separate 2 x 2 μm sections from each sheared membrane. Puncta were automatically counted with FIJI using the ‘Analyse Particles’ feature where puncta were defined as between 5–50 pixels in size. Automatic counts were manually curated to remove rings recognized as puncta. Ring structures were counted manually. All data is expressed as the number of structures per μm^2^. The mean ± the standard error is plotted.

### Image alignment

STORM-SEM overlays were generated by transforming *d*STORM images to align with their SEM equivalents. Transformations were performed with FIJI using the landmark correspondence plugin [54]

### SEM knob diameter

Knob diameter was measured using FIJI. Sample brightness was plotted from a line across each knob. The diameter of each knob was recorded as the distance between the base of each side of its brightness peak. See S1C Fig for illustration.

### Localization event counts

To determine the number of localization events per puncta or ring structures a custom Python script was created. Squares (200 x 200 nm) were placed around either isolated puncta or isolated ring structures on 23 cell membranes from 22 and 24 h post-invasion cultures. Using the squares coordinates, we counted the number of localization detections from the raw Tracked.txt tables corresponding to each punctum or ring structure, using a custom Python script (Juan Nunez-Iglesias, 2018, August 27; Count doughnuts (ring structures) Version 0.1.0. Zenodo. http://doi.org/10.5281/zenodo.1403937) depending on the pandas [55], matplotlib [56] and seaborn [57] libraries.

### Statistical analysis

Statistical analysis and graphs were generated using Prism 5 (GraphPad). Unpaired t tests were performed where specified (see figure legends). *P* values of < 0.05 (*), < 0.01 (**) and < 0.001 (***) were considered statistically significant.

## Supporting information

S1 Fig *d*STORM and SEM analysis of knob morphology. (A) Schematic diagram illustrating the preparation of membranes by hypotonic shearing, for *d*STORM and SEM imaging. (B) DIC (top) and widefield fluorescence (bottom) images of sheared trophozoite-infected RBC membranes labelled with anti-KAHRP (mAb89) and Alexa-647 antibodies. Scale bar: 10 μm. (C) Annotated examples illustrating how knob diameter measurements were performed for SEM and *d*STORM images. (D) Measurements of knobs and puncta from SEM (grey) and *d*STORM of sheared membranes (n = 10 and 5 cells for SEM and *d*STORM respectively). Data is presented as the mean ± SEM (unpaired t-test, NS p = 0.3). (E) A representative selection of the ring structures used for measuring the distances between puncta and for use in the localization detection measurements are shown. Scale bar: 100 nm. (F,G) The number of fluorescence events detected by *d*STORM within 200 x 200 nm regions drawn around individual anti-KAHRP labelled puncta and ring structures was counted. Puncta (bin size = 20) and ring structures (bin size = 50) were grouped and plotted by the number of fluorescence detections they contain.
(TIF)

S2 Fig Quantification of knob density at the RBC surface. (A) Whole CS2-infected RBCs at 16, 20, 24 and 30 h post-invasion were imaged by SEM to confirm the timing of knob appearance at the RBC membrane. Scale bar: 1 μm. (B) Quantification of the numbers of knobs at the infected RBC surface at 16, 20, 24 and 30 h post-invasion as imaged by whole cell SEM (n = 27, 26, 22 and 13 cells respectively). Data is represented as the mean ± SEM (unpaired t-test, ** p ≤ 0.005, *** p ≤ 0.0001).
(TIF)

S3 Fig Cytochalasin D prevents the formation of disk-like knobs. (A) CS2-infected RBCs were synchronized to a 2 h window and subjected to microbead filtration at 18, 20, 24 and 30 h post-invasion. Cells were treated with or without a pulse of cytochalasin D (10 μM) applied 2 h prior to filtration. Infected RBCs were passaged though a bed of microbeads and the parasitemia in the flow-through was assessed. Data represent four separate experiments each in triplicate (unpaired t-test, ns = not significant, ** p < 0.001). (B) CS2-infected RBCs were treated with or without cytochalasin D (10 μM, from 16 h post-invasion) and analyzed at 22 h post-invasion (hpi) or 8 h after removal of drug (Cyto D 8h rec) from the 22 h post-invasion group (*i*.*e*. at 30 h post-invasion). Membranes from these cells were sheared and labelled with anti-KAHRP (mAb89) and anti-mouse Alexa-647 secondary antibodies. *d*STORM, SEM and STORM-SEM images are shown as representative 1 x 1 μm sections. Scale bar: 500 nm.
(TIF)

S4 Fig Analysis of Giemsa-stained smears following cytochalasin D treatment. (A) Representative images from Giemsa-stained smears from cytochalasin D treated and untreated parasite infected RBCs. Three representative cells from each treatment are shown. Scale bar: 5 μm. (B) Measurements of cell size from images of Giemsa-stained smears. CS2-infected RBCs were treated with or without cytochalasin D (10 μM, from 16–22 h post-invasion) and examined immediately or following 8 h recovery after removal of drug at 22 h post-invasion. Data is presented as the mean area ± SEM. A minimum of 86 cells from 3 separate experiments were measured for each group (unpaired t-test, NS p = 0.05–0.9). (C) Analysis of parasitaemia in the next lifecycle after removal of cytochalasin D at 22 and 30 h post-invasion. Data is presented as the mean ± SEM, control and 16–22 h Cyto D, n = 7. 16–30 h Cyto D, n = 3. (Unpaired t-test, NS p = 0.98, **** p < 0.0001).
(TIF)

S5 Fig C-terminal truncation of the KAHRP spectrin-binding domain. (A) Schematic representation of the KAHRP truncations expressed in the transfected lines illustrating the binding regions of anti-KAHRP (mAb89: amino acids 424–539 and mAb18.2: amino acids 282–362). (B) Western blotting of saponin-lysed pellets of CS2 KAHRP truncation transfectants, probed with anti-KAHRP (mAb18.2). Cell lines were analysed separately due to varying expression levels of the different KAHRP truncations. (C) Immunofluorescence microscopy of fixed infected RBCs labelled with anti-spectrin (red) and anti-KAHRP (mAb18.2, green). The nuclei are stained with DAPI. Merges of the three labels are shown on the right. Scale bar: 1 μm. (D) Image quantification showing the levels of truncated KAHRP present relative to the spectrin control. The mean ± SEM values are plotted. 22 cells from each truncation from 3 experiments were analysed.
(TIF)

S6 Fig Knob density analysis of KAHRP truncation parasites. (A) Whole cell SEM of wildtype CS2 and the KAHRP truncation parasites showing their knob phenotypes. Scale bar: 1 μm. (B) Zoomed sections of SEM images of infected RBCs. Scale bar: 500 nm. (C) Quantification of the number (mean ± SEM) of fluorescently-labelled rings/baubles (left) and puncta (right) from *d*STORM imaging of CS2 and K530 membranes (n = 13 and 16 membranes, respectively) (unpaired t-test, ** p = 0.036, *** p = 0.0001, ns = not significant). (D) Number of knobs (mean ± SEM) on the surface of 30 h post-invasion CS2- and K530-infected RBCs (n = 13 and 16 cells, respectively).
(TIF)

S7 Fig Analysis of KAHRP location in truncation parasites. (A) *d*STORM imaging of sheared membranes from CS2 wildtype and K530 truncation parasites at 30 and 40 h post-invasion. Membranes were labelled with anti-KAHRP (mAb18.2) and anti-mouse Alexa-647 secondary antibodies. Full sheared membranes are displayed in the top panel and 1 x 1 μm zoomed images are shown below. Scale bars: 1 μm (top), 500 nm (bottom). (B) Quantification of the number (mean ± SEM) of fluorescently-labelled rings/baubles (left) and puncta (right) from *d*STORM imaging of CS2 and K530 membranes (n = 13 and 16 membranes, respectively) (unpaired t-test, ** p = 0.0037, ns = not significant).
(TIF)

S8 Fig Analysis of spiral morphology in KAHRP truncation parasites. Projections from electron tomograms of negatively-stained preparations of schizont-infected RBCs from the CS2, K530 and K405 lines. Five examples are presented showing the morphology of the full and fragmented knobs. The spiral structures were segmented manually and are shown as an overlay in the bottom panel. Scale bar: 20 nm.
(TIF)

S9 Fig Quantification of spiral morphology in KAHRP truncation parasites. (A) Individual graphs showing the distribution of empty knobs, full and fragments (Frag) spirals in wildtype CS2 and the K530 and K405 truncation mutants. The mean ± SEM is plotted. The individual counts are shown in the tables on the right. Tomo = Electron tomogram. The values in the total column are used to generate the graph in Fig 5D. (B,C,D) The diameters of spirals (B), the lengths of spirals (C) and widths of the electron-dense knobs (D) were measured from electron tomograms of negative-stained preparations of schizont-infected RBCs. Measurements were taken from 10 knobs for each parasite line (unpaired t-test, ** p = 0.006, *** p = 0.0009). Data is presented as the mean ± SEM. The data used to generate these graphs are shown in the tables on the right.
(TIF)

S10 Fig Proximity of PfEMP1A-GFP and knobs on sheared membranes. (A) PfEMP1A-GFP distribution on sheared membranes 20 h post-invasion. Membranes were labelled with anti-GFP and anti-mouse Alexa-647 secondary antibodies. Scale bar: 1 μm. (B) Examples of PfEMP1A-GFP foci imaged by STORM-SEM, at, adjacent to and away from knobs are illustrated in magnified (300 x 300 nm) regions. A 50 nm radius (blue circle) and 100 nm radius (yellow circle) is shown from the center of the knob (white dot). (C) SEM and STORM-SEM of sheared membranes prepared from RBCs infected with PfEMP1A-GFP transfectants at 20, 24 and 30 h post-invasion. Representative 2 x 2 μm sections highlight the distribution of PfEMP1A-GFP in the membrane. Scale bar: 500 nm.
(TIF)

S11 Fig A model for aberrant knob formation in KAHRP mutant cells. CS2: Wildtype KAHRP forms a ring, organized and scaffolded through spectrin binding. K530: Truncation of the 3’ repeat region results in an aberrant KAHRP arrangement and spiral fragmentation. K405: Partial truncation of the KAHRP spectrin-binding domain results in impaired membrane skeleton binding, KAHRP clustering and severe spiral fragmentation.
(TIF)
