# Supplementary figures and images for: The knob protein KAHRP assembles into a ring-shaped structure that underpins virulence complex assembly
Source: PLoS Pathog. 2019 May 9;15(5):e1007761. doi: 10.1371/journal.ppat.1007761 (PMC6529015; doi:10.1371/journal.ppat.1007761)

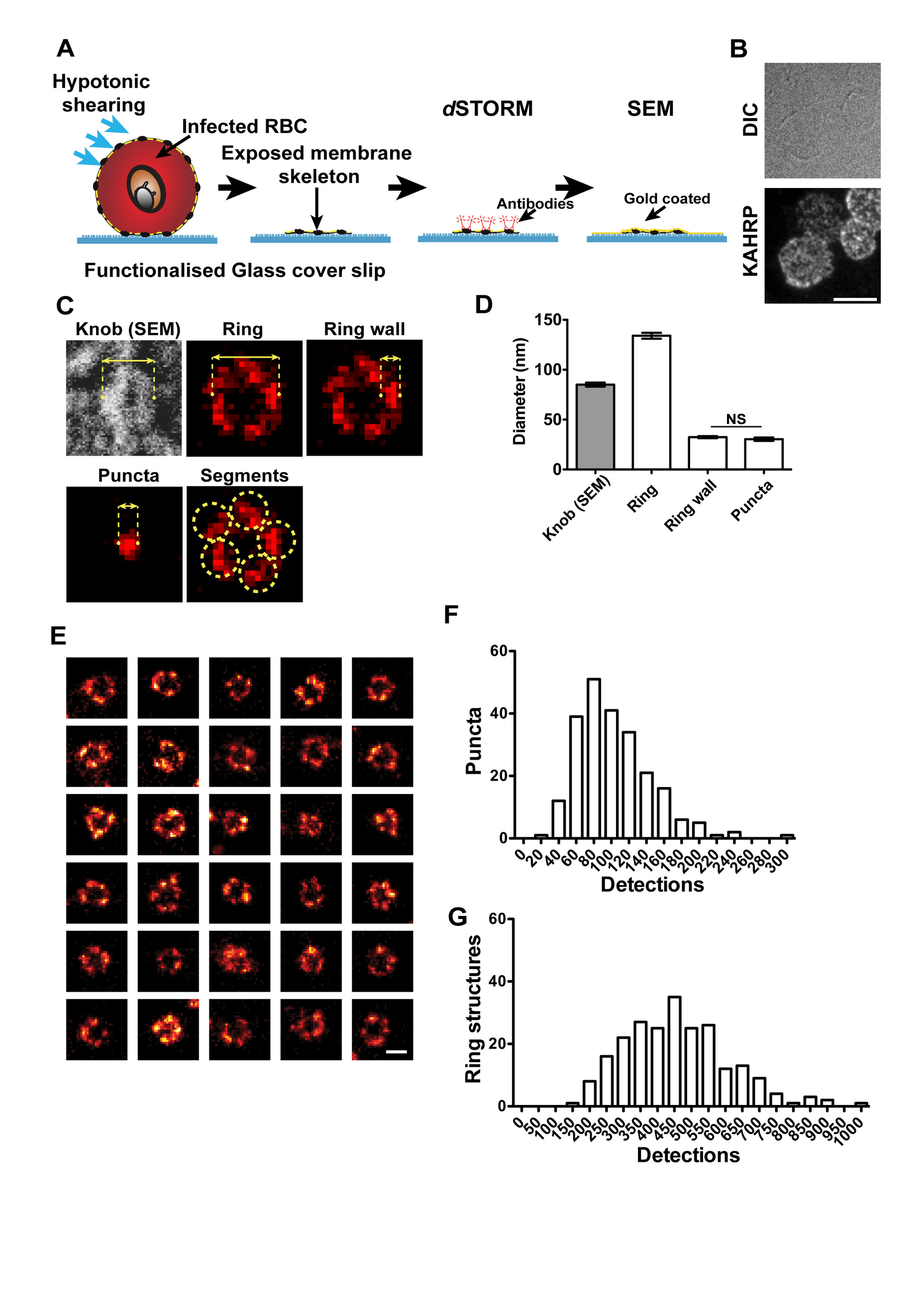

Supplement: S1 Fig — (A) Schematic diagram illustrating the preparation of membranes by hypotonic shearing, for dSTORM and SEM imaging. (B) DIC (top) and widefield fluorescence (bottom) images of sheared trophozoite-infected RBC membranes labelled with anti-KAHRP (mAb89) and Alexa-647 antibodies. Scale bar: 10 μm. (C) Annotated examples illustrating how knob diameter measurements were performed for SEM and dSTORM images. (D) Measurements of knobs and puncta from SEM (grey) and dSTORM of sheared membranes (n = 10 and 5 cells for SEM and dSTORM respectively). Data is presented as the mean ± SEM (unpaired t-test, NS p = 0.3). (E) A representative selection of the ring structures used for measuring the distances between puncta and for use in the localization detection measurements are shown. Scale bar: 100 nm. (F,G) The number of fluorescence events detected by dSTORM within 200 x 200 nm regions drawn around individual anti-KAHRP labelled puncta and ring structures was counted. Puncta (bin size = 20) and ring structures (bin size = 50) were grouped and plotted by the number of fluorescence detections they contain. (TIF) [file ppat.1007761.s001.tif]

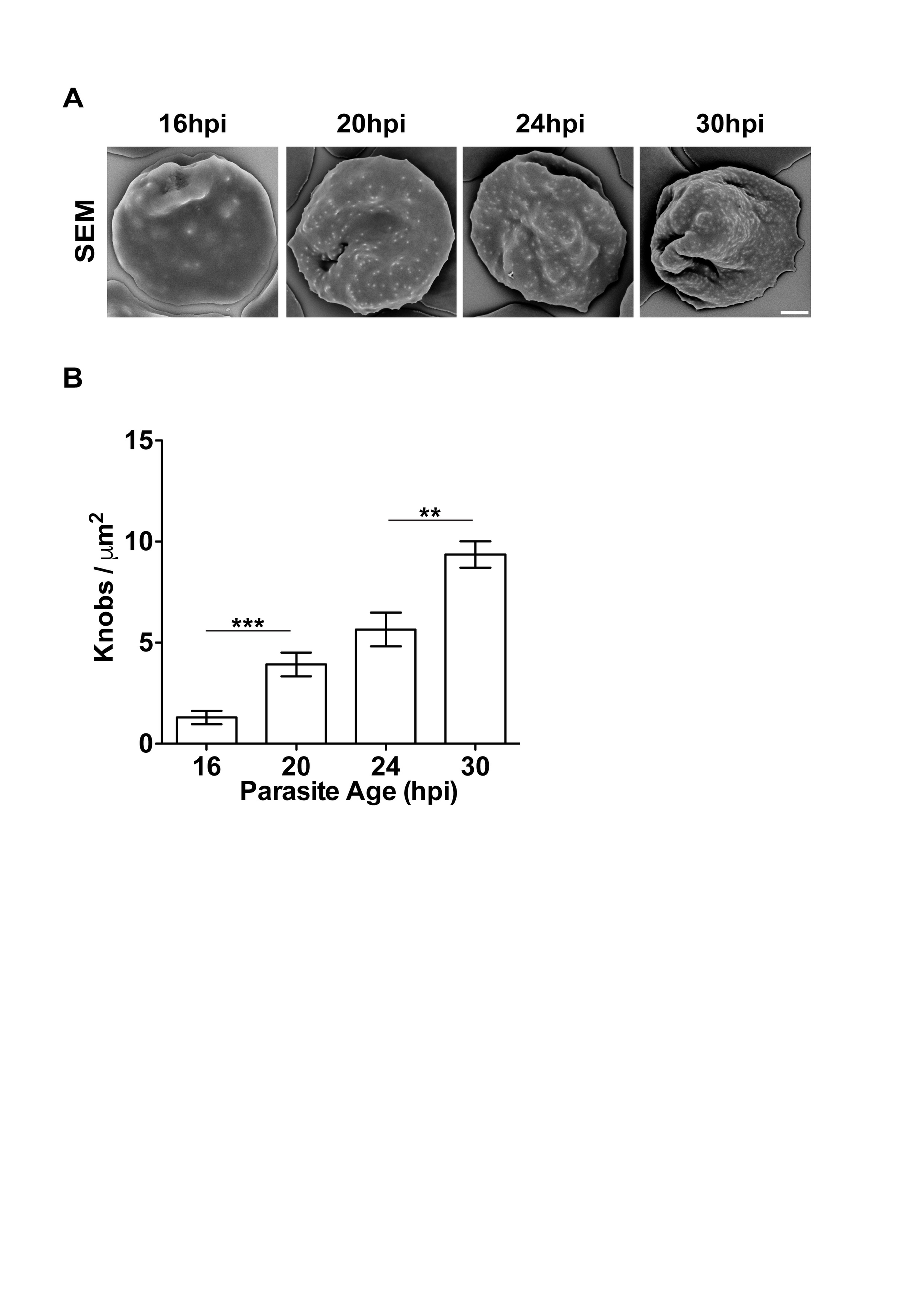

Supplement: S2 Fig — (A) Whole CS2-infected RBCs at 16, 20, 24 and 30 h post-invasion were imaged by SEM to confirm the timing of knob appearance at the RBC membrane. Scale bar: 1 μm. (B) Quantification of the numbers of knobs at the infected RBC surface at 16, 20, 24 and 30 h post-invasion as imaged by whole cell SEM (n = 27, 26, 22 and 13 cells respectively). Data is represented as the mean ± SEM (unpaired t-test, ** p ≤ 0.005, *** p ≤ 0.0001). (TIF) [file ppat.1007761.s002.tif]

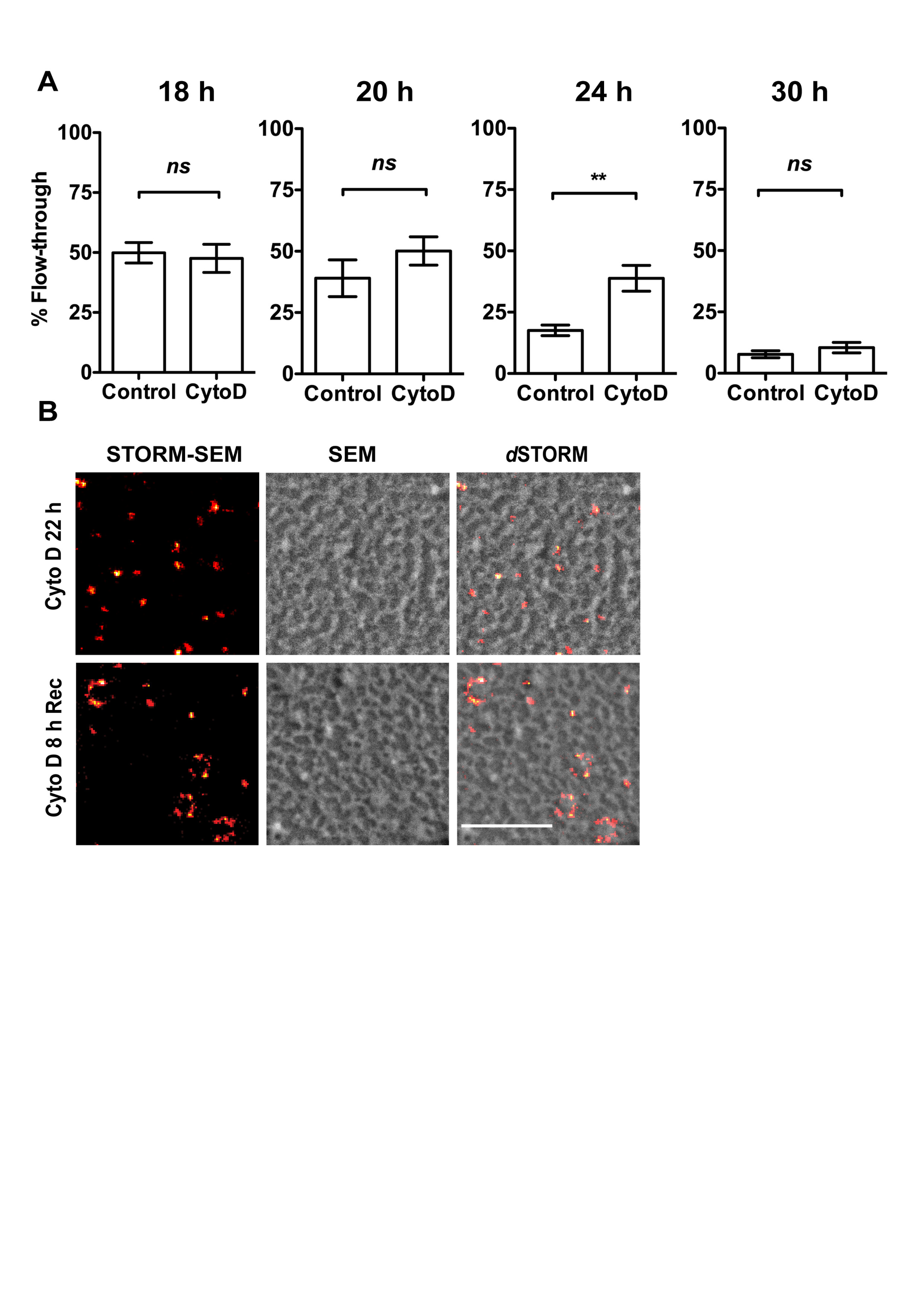

Supplement: S3 Fig — (A) CS2-infected RBCs were synchronized to a 2 h window and subjected to microbead filtration at 18, 20, 24 and 30 h post-invasion. Cells were treated with or without a pulse of cytochalasin D (10 μM) applied 2 h prior to filtration. Infected RBCs were passaged though a bed of microbeads and the parasitemia in the flow-through was assessed. Data represent four separate experiments each in triplicate (unpaired t-test, ns = not significant, ** p < 0.001). (B) CS2-infected RBCs were treated with or without cytochalasin D (10 μM, from 16 h post-invasion) and analyzed at 22 h post-invasion (hpi) or 8 h after removal of drug (Cyto D 8h rec) from the 22 h post-invasion group (i.e. at 30 h post-invasion). Membranes from these cells were sheared and labelled with anti-KAHRP (mAb89) and anti-mouse Alexa-647 secondary antibodies. dSTORM, SEM and STORM-SEM images are shown as representative 1 x 1 μm sections. Scale bar: 500 nm. (TIF) [file ppat.1007761.s003.tif]

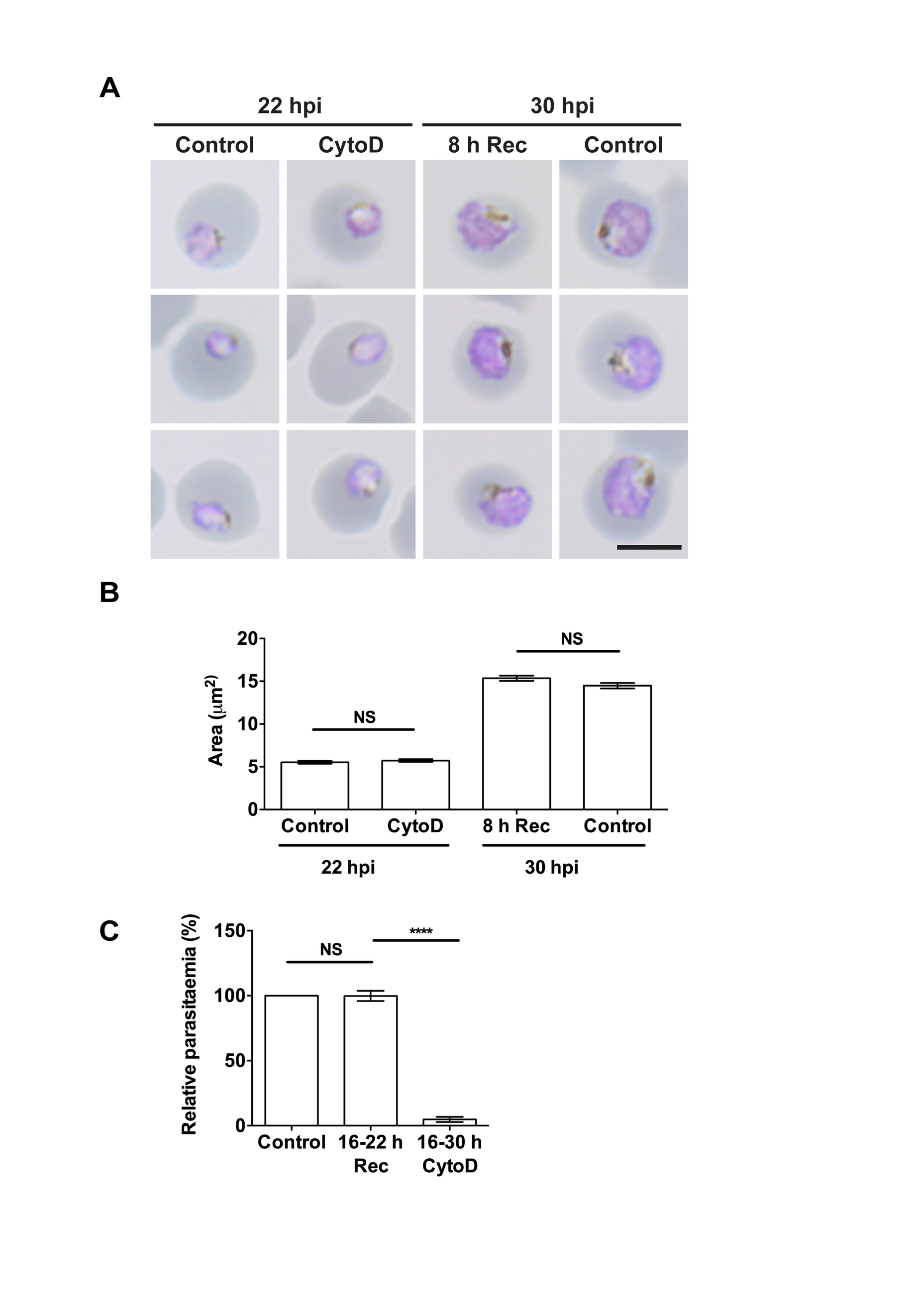

Supplement: S4 Fig — (A) Representative images from Giemsa-stained smears from cytochalasin D treated and untreated parasite infected RBCs. Three representative cells from each treatment are shown. Scale bar: 5 μm. (B) Measurements of cell size from images of Giemsa-stained smears. CS2-infected RBCs were treated with or without cytochalasin D (10 μM, from 16–22 h post-invasion) and examined immediately or following 8 h recovery after removal of drug at 22 h post-invasion. Data is presented as the mean area ± SEM. A minimum of 86 cells from 3 separate experiments were measured for each group (unpaired t-test, NS p = 0.05–0.9). (C) Analysis of parasitaemia in the next lifecycle after removal of cytochalasin D at 22 and 30 h post-invasion. Data is presented as the mean ± SEM, control and 16–22 h Cyto D, n = 7. 16–30 h Cyto D, n = 3. (Unpaired t-test, NS p = 0.98, **** p < 0.0001). (TIF) [file ppat.1007761.s004.tif]

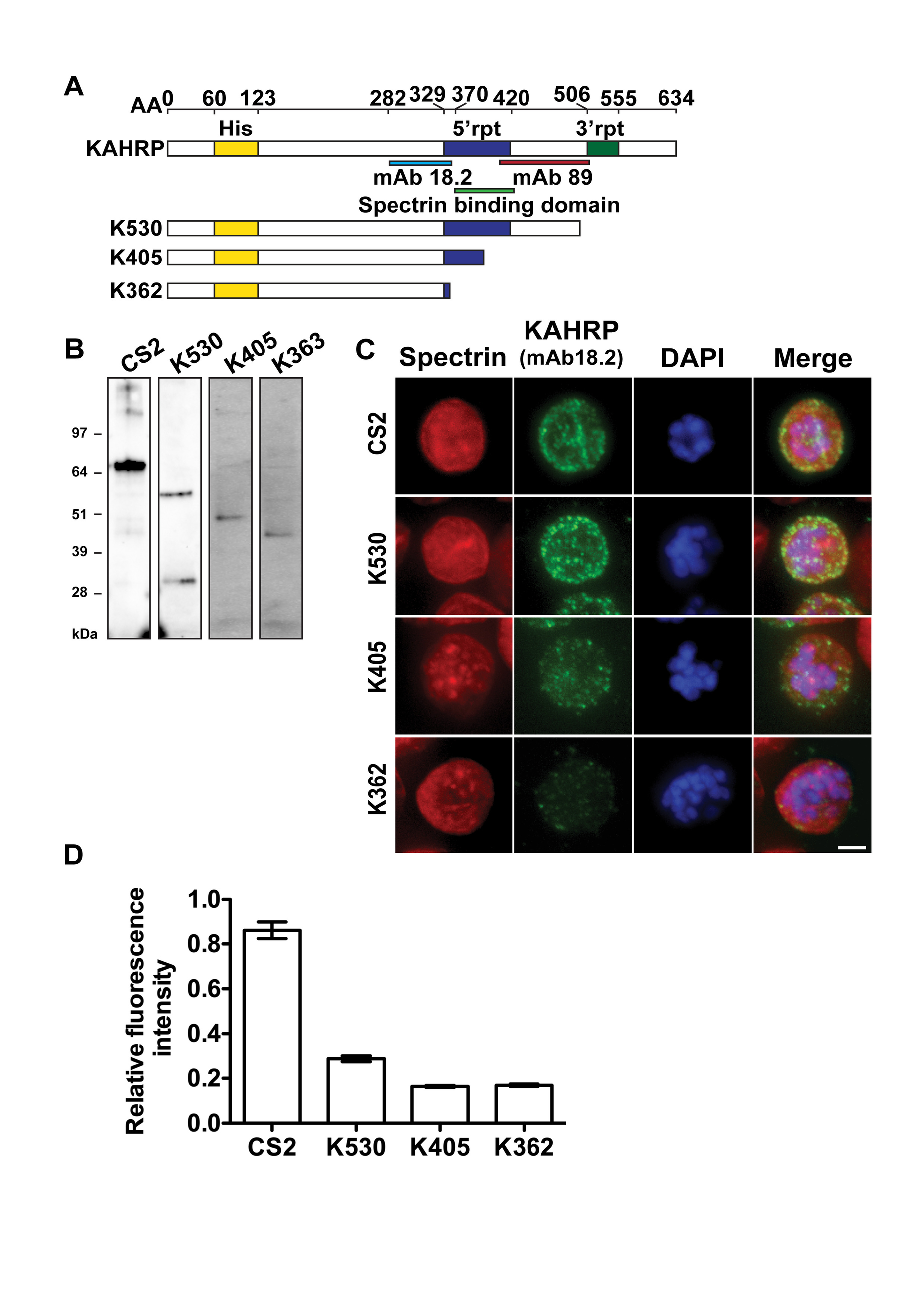

Supplement: S5 Fig — (A) Schematic representation of the KAHRP truncations expressed in the transfected lines illustrating the binding regions of anti-KAHRP (mAb89: amino acids 424–539 and mAb18.2: amino acids 282–362). (B) Western blotting of saponin-lysed pellets of CS2 KAHRP truncation transfectants, probed with anti-KAHRP (mAb18.2). Cell lines were analysed separately due to varying expression levels of the different KAHRP truncations. (C) Immunofluorescence microscopy of fixed infected RBCs labelled with anti-spectrin (red) and anti-KAHRP (mAb18.2, green). The nuclei are stained with DAPI. Merges of the three labels are shown on the right. Scale bar: 1 μm. (D) Image quantification showing the levels of truncated KAHRP present relative to the spectrin control. The mean ± SEM values are plotted. 22 cells from each truncation from 3 experiments were analysed. (TIF) [file ppat.1007761.s005.tif]

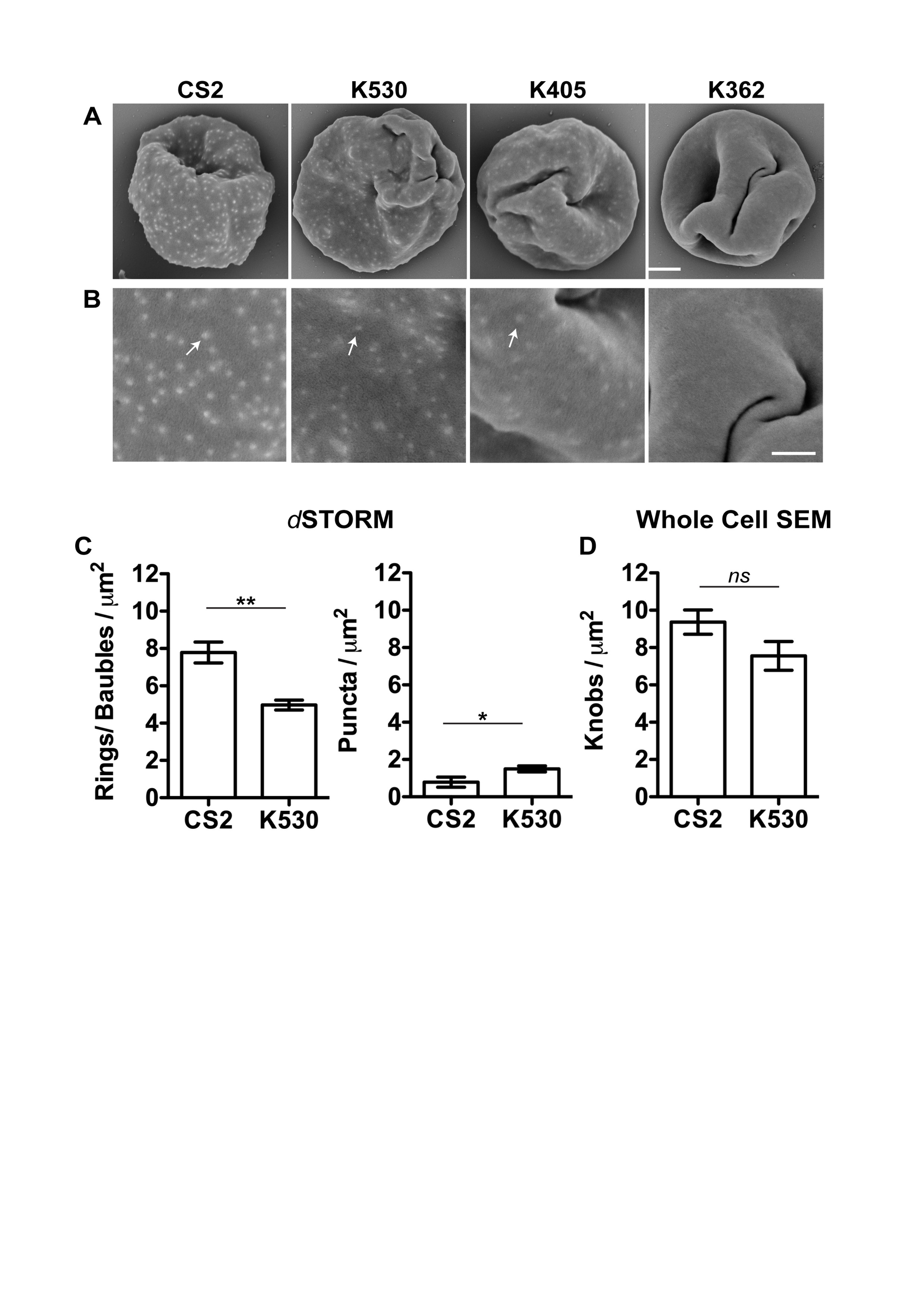

Supplement: S6 Fig — (A) Whole cell SEM of wildtype CS2 and the KAHRP truncation parasites showing their knob phenotypes. Scale bar: 1 μm. (B) Zoomed sections of SEM images of infected RBCs. Scale bar: 500 nm. (C) Quantification of the number (mean ± SEM) of fluorescently-labelled rings/baubles (left) and puncta (right) from dSTORM imaging of CS2 and K530 membranes (n = 13 and 16 membranes, respectively) (unpaired t-test, ** p = 0.036, *** p = 0.0001, ns = not significant). (D) Number of knobs (mean ± SEM) on the surface of 30 h post-invasion CS2- and K530-infected RBCs (n = 13 and 16 cells, respectively). (TIF) [file ppat.1007761.s006.tif]

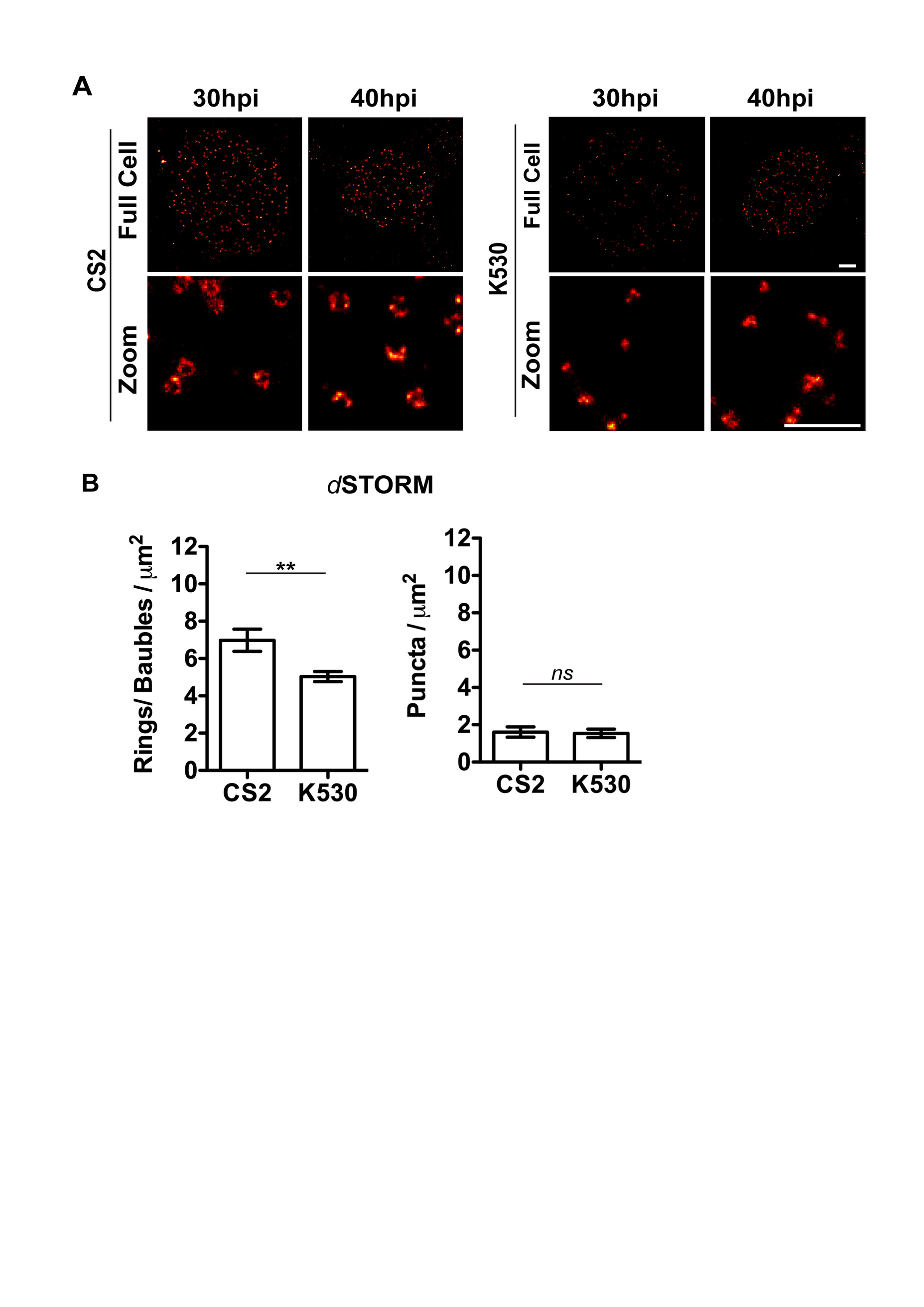

Supplement: S7 Fig — (A) dSTORM imaging of sheared membranes from CS2 wildtype and K530 truncation parasites at 30 and 40 h post-invasion. Membranes were labelled with anti-KAHRP (mAb18.2) and anti-mouse Alexa-647 secondary antibodies. Full sheared membranes are displayed in the top panel and 1 x 1 μm zoomed images are shown below. Scale bars: 1 μm (top), 500 nm (bottom). (B) Quantification of the number (mean ± SEM) of fluorescently-labelled rings/baubles (left) and puncta (right) from dSTORM imaging of CS2 and K530 membranes (n = 13 and 16 membranes, respectively) (unpaired t-test, ** p = 0.0037, ns = not significant). (TIF) [file ppat.1007761.s007.tif]

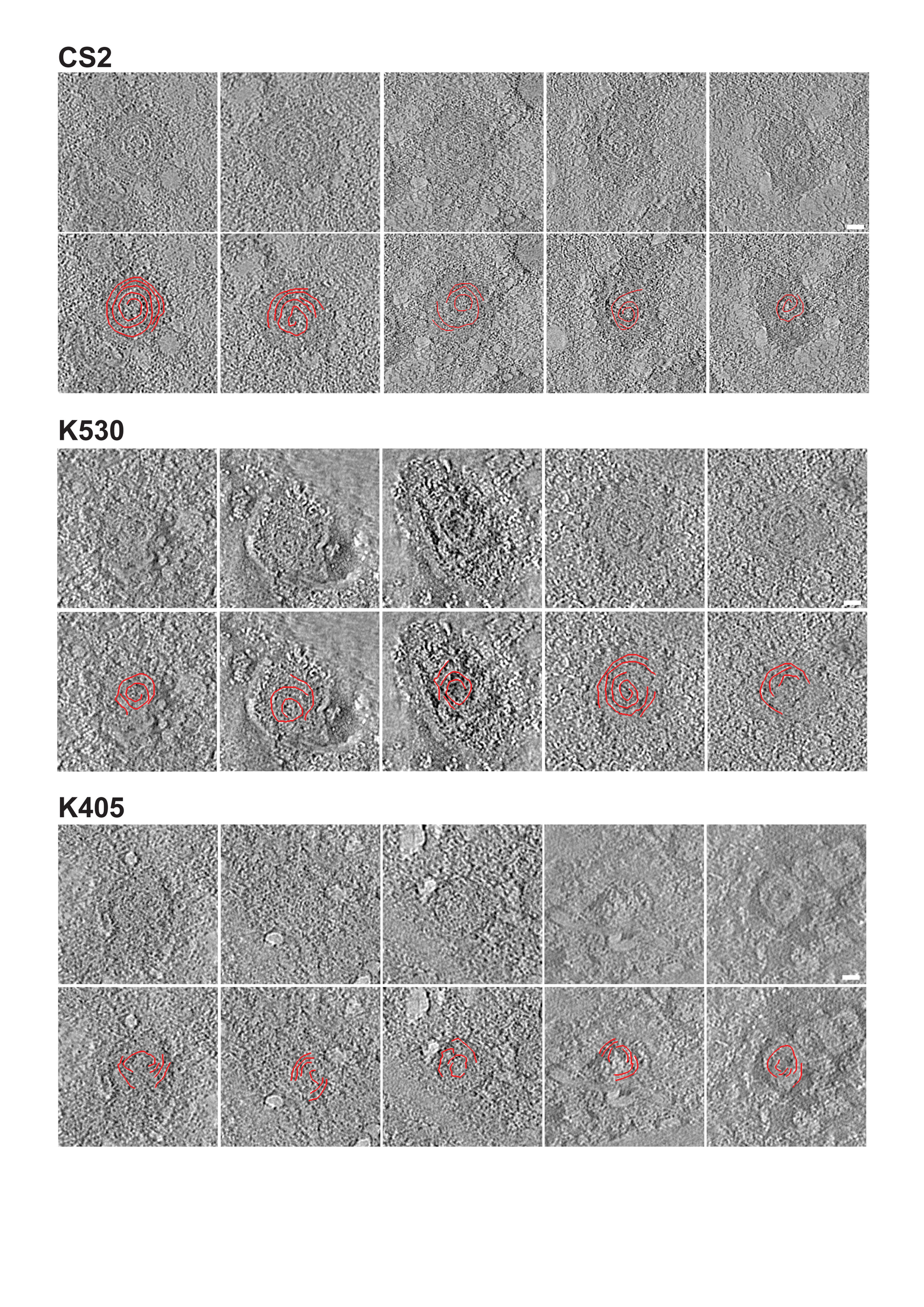

Supplement: S8 Fig — Projections from electron tomograms of negatively-stained preparations of schizont-infected RBCs from the CS2, K530 and K405 lines. Five examples are presented showing the morphology of the full and fragmented knobs. The spiral structures were segmented manually and are shown as an overlay in the bottom panel. Scale bar: 20 nm. (TIF) [file ppat.1007761.s008.tif]

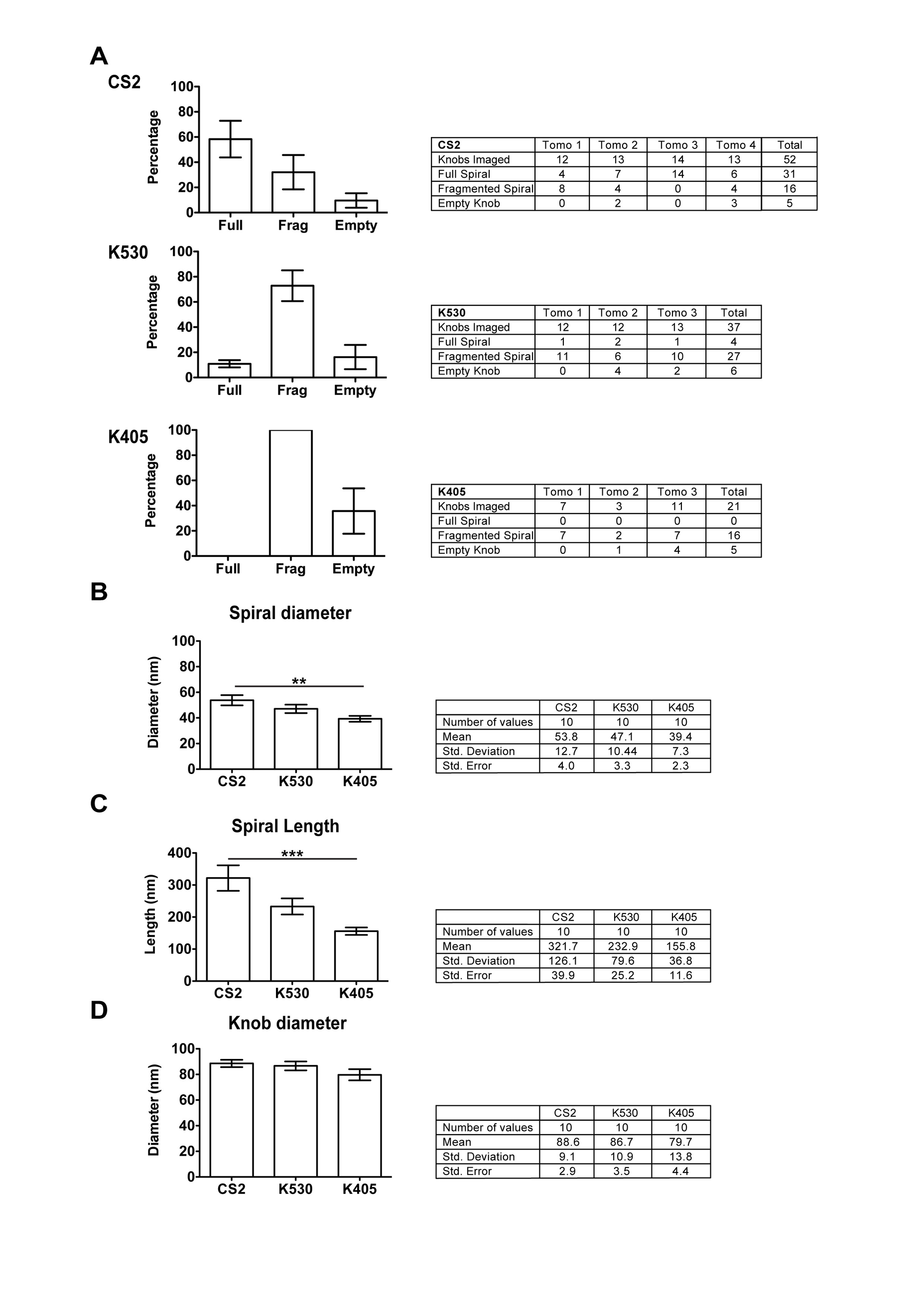

Supplement: S9 Fig — (A) Individual graphs showing the distribution of empty knobs, full and fragments (Frag) spirals in wildtype CS2 and the K530 and K405 truncation mutants. The mean ± SEM is plotted. The individual counts are shown in the tables on the right. Tomo = Electron tomogram. The values in the total column are used to generate the graph in Fig 5D. (B,C,D) The diameters of spirals (B), the lengths of spirals (C) and widths of the electron-dense knobs (D) were measured from electron tomograms of negative-stained preparations of schizont-infected RBCs. Measurements were taken from 10 knobs for each parasite line (unpaired t-test, ** p = 0.006, *** p = 0.0009). Data is presented as the mean ± SEM. The data used to generate these graphs are shown in the tables on the right. (TIF) [file ppat.1007761.s009.tif]

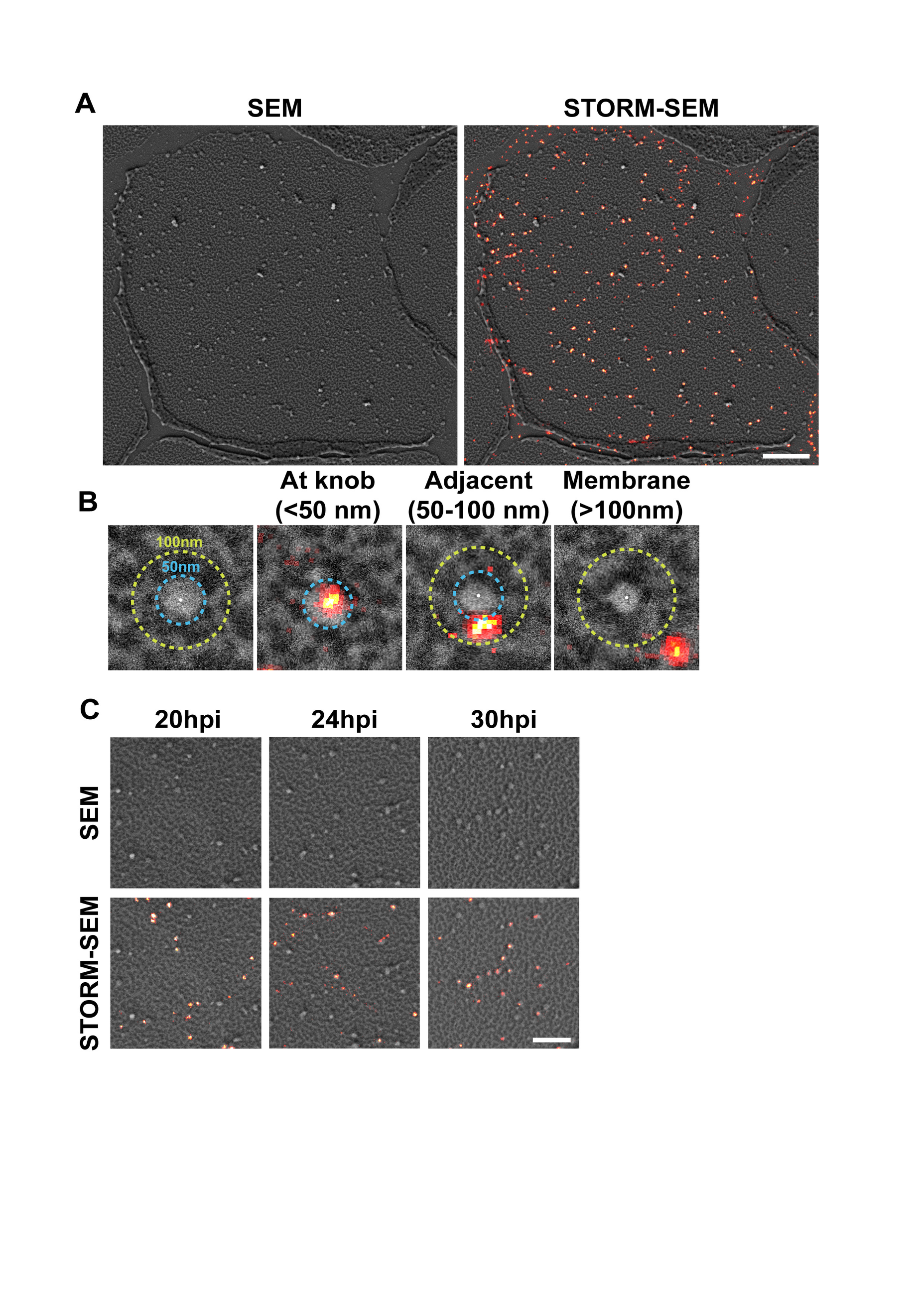

Supplement: S10 Fig — (A) PfEMP1A-GFP distribution on sheared membranes 20 h post-invasion. Membranes were labelled with anti-GFP and anti-mouse Alexa-647 secondary antibodies. Scale bar: 1 μm. (B) Examples of PfEMP1A-GFP foci imaged by STORM-SEM, at, adjacent to and away from knobs are illustrated in magnified (300 x 300 nm) regions. A 50 nm radius (blue circle) and 100 nm radius (yellow circle) is shown from the center of the knob (white dot). (C) SEM and STORM-SEM of sheared membranes prepared from RBCs infected with PfEMP1A-GFP transfectants at 20, 24 and 30 h post-invasion. Representative 2 x 2 μm sections highlight the distribution of PfEMP1A-GFP in the membrane. Scale bar: 500 nm. (TIF) [file ppat.1007761.s010.tif]

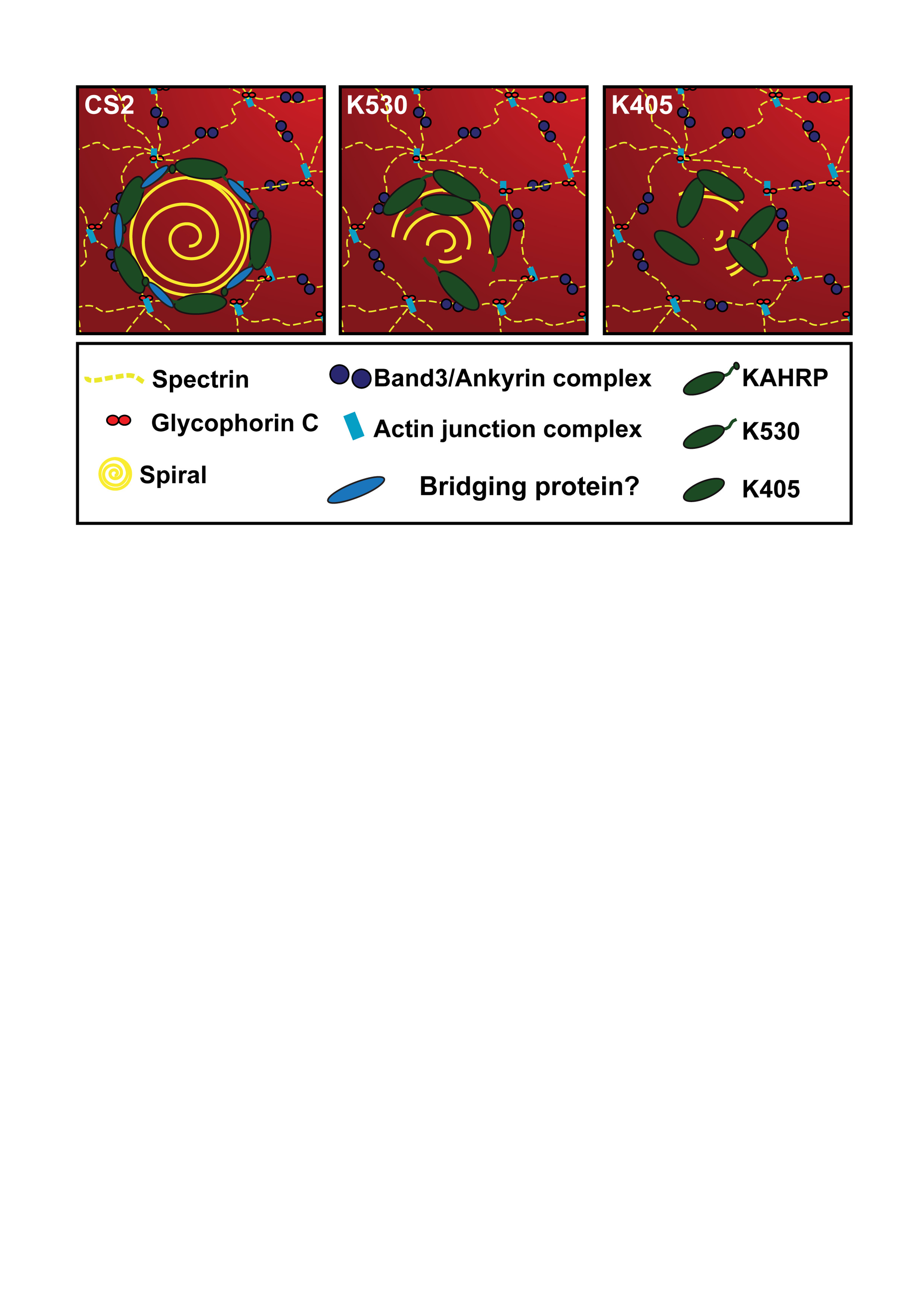

Supplement: S11 Fig — CS2: Wildtype KAHRP forms a ring, organized and scaffolded through spectrin binding. K530: Truncation of the 3’ repeat region results in an aberrant KAHRP arrangement and spiral fragmentation. K405: Partial truncation of the KAHRP spectrin-binding domain results in impaired membrane skeleton binding, KAHRP clustering and severe spiral fragmentation. (TIF) [file ppat.1007761.s011.tif]
